# Programmable 3D Microfluidics Reveals Motility‐Driven Antibiotic Resistance Evolution in Gradients

**DOI:** 10.1002/smll.75309

**Published:** 2026-08-18

**Authors:** Xiaobo Li, Jianan Yin, Yanqing Song, Andrew Glidle, William Sloan, He Huang, Maggie Cusack, Cindy J. Smith, Huabing Yin

**Affiliations:** ^1^ James Watt School of Engineering Advanced Research Centre (ARC) University of Glasgow Glasgow UK; ^2^ School of Synthetic Biology and Biomanufacturing State Key Laboratory of Synthetic Biology Tianjin Key Laboratory of Biological and Pharmaceutical Engineering Tianjin University Tianjin China; ^3^ Munster Technological University Cork Ireland

**Keywords:** antibiotic resistance, gradients, microfluidics, migration, motility, single cells

## Abstract

Antibiotic resistance has become a serious threat to global health. It frequently emerges in real‐world environments such as biofilms, where antibiotic gradients impose selective pressures that drive bacterial adaptation. However, the mechanisms underlying resistance development remain poorly understood, largely due to challenges in experimentally controlling confounding parameters. Here, we report a 3D microfluidic platform that enables real‐time monitoring of bacterial dynamics under well‐defined antibiotic gradients, along with programmable cell retrieval to identify key factors in resistance development. We show that motile *Escherichia coli* migrated randomly within the gradients regardless of antibiotic class (i.e., gentamicin, ciprofloxacin, and ampicillin). Resistance emerged only when a critical mass of bacteria sustained migration through the gradient for a sufficiently duration, revealing a previously unrecognized migration‐duration threshold for adaptive evolution. Whole‐genome resequencing revealed gene mutations in the resistant cells, including mutations in ATP synthase‐related genes (i.e., *atpG*, *atpD*, and *atpF*). A trade‐off between resistance and cell growth was also observed. Similar phenomena were observed in other motile bacteria species, including *Serratia marcescens*. Overall, we demonstrate that cell motility is a critical determinant of resistance development in heterogeneous antibiotic landscapes. Targeting cell motility and growth could offer effective strategies to combat antibiotic resistance in motile bacteria.

## Graphical abstract

Using a programmable microfluidic platform, this study shows that bacterial motility is crucial for resistance development in heterogeneous antibiotic landscapes. Motile *Escherichia coli* randomly migrates along antibiotic gradients, and sufficient movement is required for resistance to emerge. Targeting motility may offer a promising strategy for combating antibiotic resistance in motile bacteria.

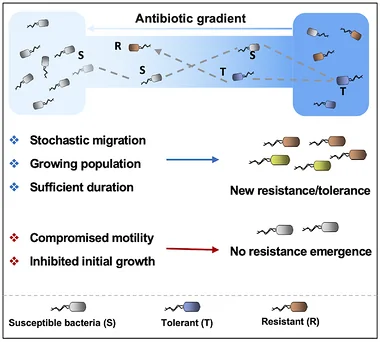

## 1 Introduction

Antimicrobial resistance (AMR) is threatening global health and the economy at an unprecedented speed [1]. However, the development of new antimicrobial compounds is slow and is outpaced by the emergence of new resistance [2, 3, 4]. An in‐depth understanding of mechanisms underlying resistance evolution is critical to combating the AMR crisis [5, 6].

In real‐world applications, antibiotic treatments often result in spatially heterogeneous concentration gradients, ranging from zero to lethal inhibitory levels. This leads to some bacteria exposed to sub‐inhibitory concentrations, which can contribute to the development of antibiotic resistance [7, 8]. For motile bacteria, some studies show that gradients facilitated their adaptive resistance to the antibiotics [9, 10, 11, 12, 13], while others show the opposite [14, 15]. Notably, cell motility was not controlled in these studies, but was convoluted with cell growth and colonization, which may contribute to the observed discrepancies [9, 11, 14, 15]. Recently, a quantitative model revealed cell motility can both accelerate and limit bacterial adaptation in antibiotic landscapes, depending on the motility regime cells are in [16]. However, classifying these regimes requires well‐characterized cell motility, growth, and the geometry of the antibiotic landscape; these often change during the emergence of antibiotic resistance. The capability to study dynamic responses of bacteria with precisely characterized properties is essential for unravelling resistance evolution in heterogeneous antibiotic landscapes.

Here, a programmable microfluidic platform was employed to evaluate the response of motile bacterial cells in antibiotic gradients (Figure 1a). *E. coli* was chosen as the model organism, as resistant *E. coli* has been listed by the WHO as one of the highest‐priority critical pathogens in greatest need of new antibiotics [17]. The microfluidic platform enables in situ monitoring of individual bacterial movement and the isolation, at any time, of bacteria that have migrated to the antibiotic reservoir for further off‐chip characterization of their susceptibility and resistance profiles (Figure 1b). Using whole‐genome resequencing, we identified unique resistance mechanisms in *E. coli* arising from this adaptive process. Additionally, we evaluated the effects of key factors (including bacterial motility, migration duration, cell growth, and population density) on bacterial adaptation within antibiotic gradients (Figure 1c). Furthermore, this motility‐driven resistance development under antibiotic gradients was also observed in other motile bacterial species (e.g., *Serratia marcescens*). Overall, our platform provides a powerful tool to evaluate bacterial adaptation and resistance development, revealing the importance of sufficient migration across antibiotic gradients in the evolution of antibiotic resistance.

**FIGURE 1:**
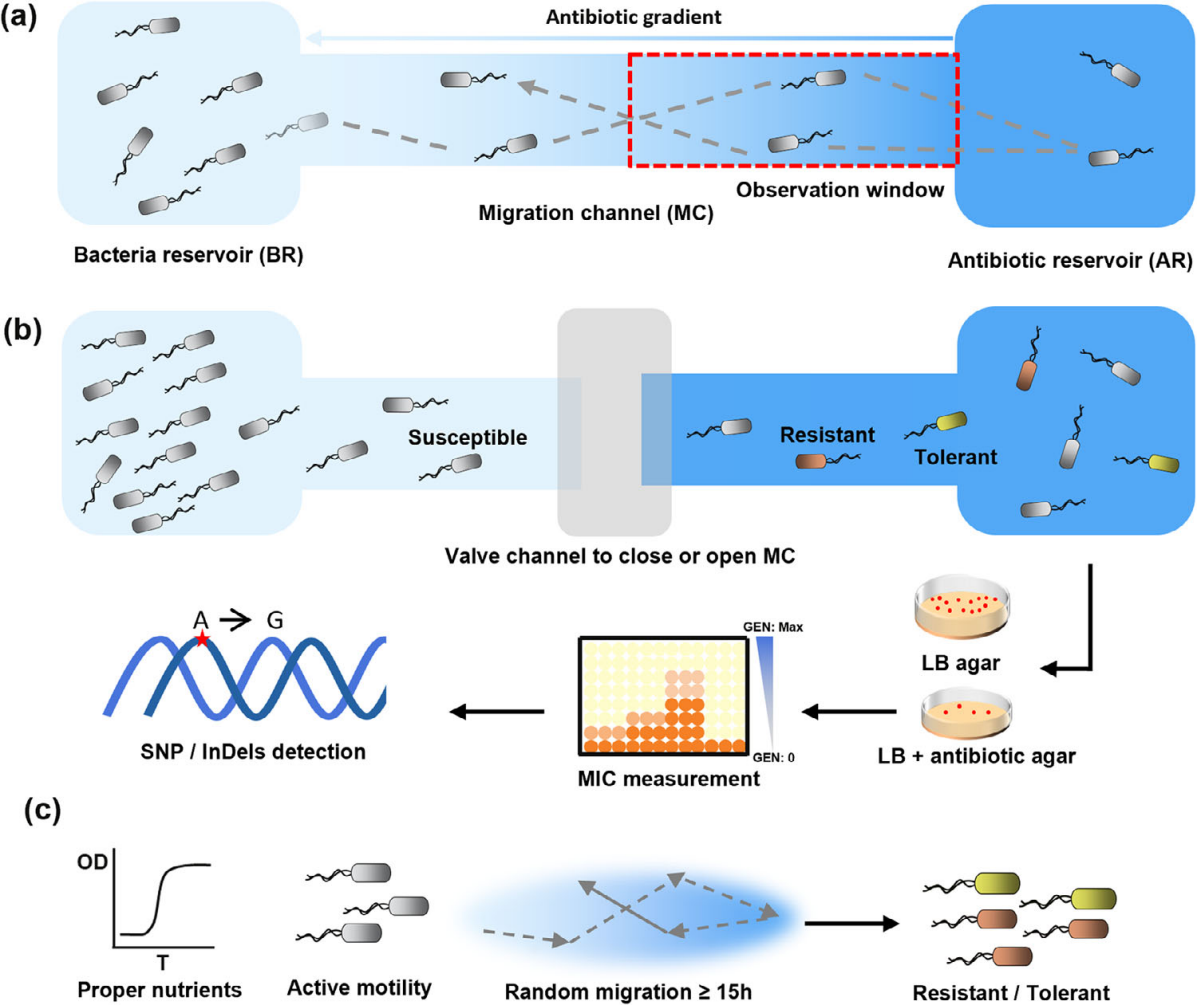
Overview of the microfluidic system and study design. (a) Responses of motile *E. coli* exposed to antibiotic gradients were tracked at the observation windows (red dotted frame). (b) After exposure for a specified period, the migration channel was closed using the on‐chip valve to retrieve bacteria that had migrated into the antibiotic reservoir for off‐chip characterization. Retrieved cells were cultured on agar plates and evaluated for their resistance profiles. Selected resistant strains underwent whole‐genome sequencing to identify mutations. (c) Key factors influencing resistance development, including nutrient availability, motility, and migration duration, were systematically investigated. BR: bacteria reservoir. AR: antibiotic reservoir. MC: migration channel. GEN: gentamicin.

## 2 Results

### 2.1 Overview of the Platform and Antibiotic Gradients

The 3D microfluidic platform was recently developed in house [18], which not only maintains a highly stable gradient along the migration channel between the sink and source reservoirs (hereafter referred to as the bacteria reservoir and antibiotic reservoir) over a long period (> 20 h), but also enables programmable delivery of samples and retrieval of bacteria off‐chip at the user‐defined manner.

Three antibiotics, including gentamicin (GEN, an aminoglycoside antibiotic), ciprofloxacin (CIP, a fluoroquinolone antibiotic), and ampicillin (AMP, a β‐lactam penicillin antibiotic), that are commonly used to treat Gram‐negative bacteria, were investigated. The minimum inhibitory concentrations (MICs) of GEN, CIP, and AMP for the ancestral *E. coli* strain were measured and found to be 0.5, 0.0078, and 8 mg/L, respectively. These MICs values suggested that this *E. coli* strain is susceptible to these antibiotics based on the CLSI breakpoints [19], with the susceptible thresholds defined as MIC ≤ 2 mg/L for GEN, ≤ 0.25 mg/L for CIP, and ≤ 8 mg/L for AMP.

Most of the antibiotic concentrations in the antibiotic reservoir exceeded 1× MIC. For simplicity, gradients were denoted as fold‐MIC antibiotic. For example, a gradient formed with 100‐fold MIC of GEN in the reservoir was denoted as 100MIC GEN gradient. Here, fluorescein was used as a proxy for antibiotic gradient formation because it has a similar molecular weight and diffusion coefficient to the antibiotics used in this study [15]. Once the migration channel was opened, a stable linear fluorescein gradient was established with approximately 50 min along the migration channel (Figure S1c). The distribution of antibiotics in the migration channel is expected to closely follow the fluorescein concentration profile. Notably, antibiotic adsorption in the PDMS chip is negligible (Figure S2). Therefore, the antibiotic concentration at the observation window near the antibiotic reservoir is expected to be greater than half of the antibiotic concentration loaded into the antibiotic reservoir (Figure S1), indicating cells reaching this point have already experienced sub‐lethal antibiotic exposure.

### 2.2 *E. coli* Exhibits Random Movement in Antibiotic Gradients

Previous work has demonstrated that *Pseudomonas aeruginosa* could bias their movements toward antibiotics, inducing suicidal chemotaxis [15]. In contrast, our data did not show such a biased movement of *E. coli* under antibiotic gradients. Instead, *E. coli* randomly changed the direction of their movements with time (Figure 2a and Figure S3), no difference was observed with or without antibiotic gradients in tryptone broth (TB). Similarly, in the chemotaxis test, there was no difference in the number of swimming cells within the observation window in the presence or absence of antibiotics in chemotaxis buffer (CB) (Figure 2b). As a positive control, in the presence of a strong chemoattractant (i.e., 0.5 mm aspartic acid), over 1000 bacteria tracked per field of view (POV) within the first 60 min (Figure 2b), denoting a strong chemotaxis behavior of this strain [20].

**FIGURE 2:**
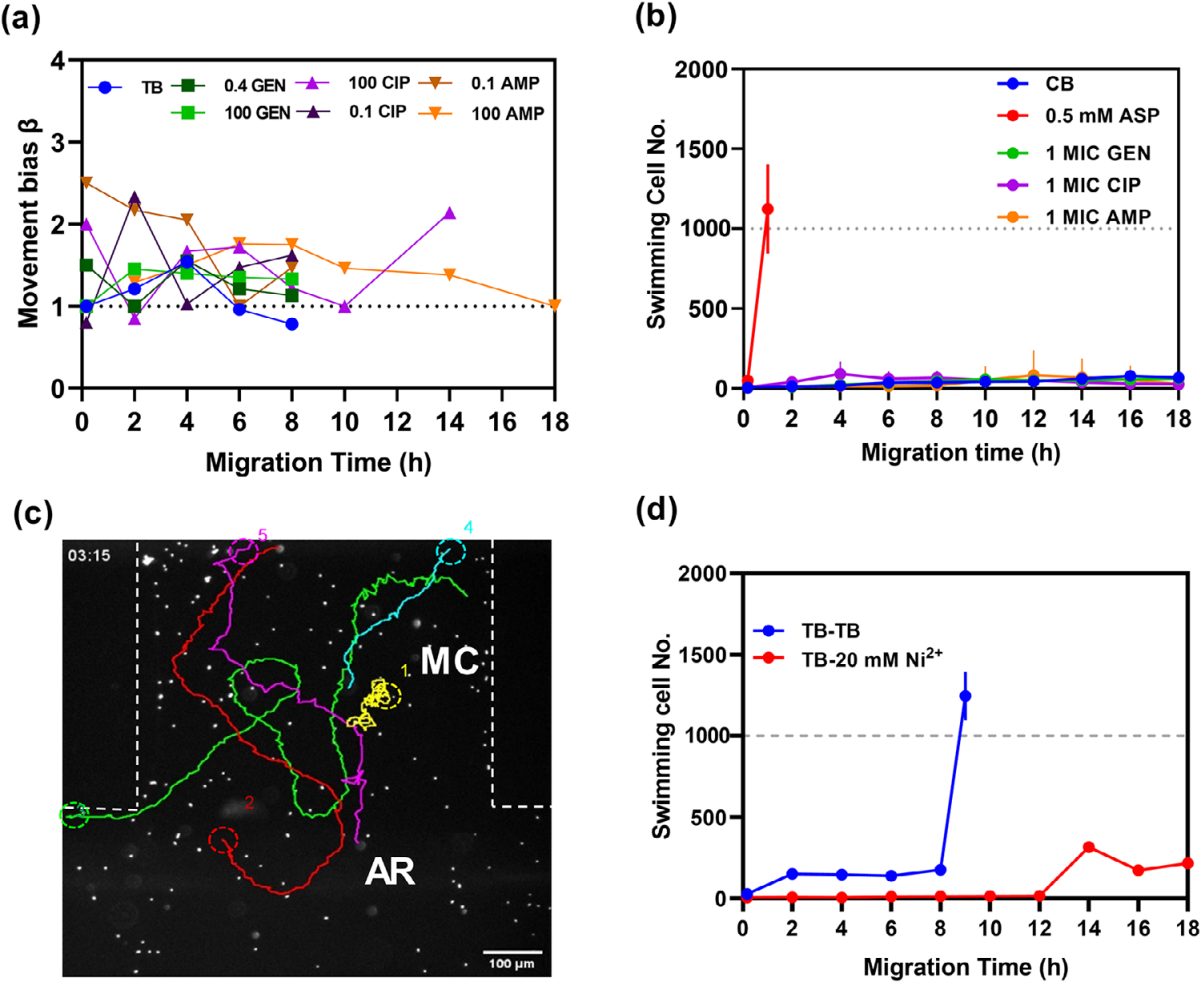
Bacteria migration under antibiotic gradients. (a) Movement bias (β) of bacteria under various gradients. Movement bias is defined as the ratio of cells swimming toward vs. away from the antibiotic source. Values preceding antibiotic numbers indicate fold‐MIC. (b) On‐chip chemotaxis assays. The number of *E. coli* cells within the observation window was tracked when the source reservoir contained CB (the control), CB containing different antibiotic concentrations, or CB supplemented with 0.5 mm ASP (the chemoattractant group). (c) Representative single‐cell swimming trajectories in the antibiotic reservoir (AR) and migration channel (MC). Each white dot indicates a cell; trajectories are colored. Dotted circles mark starting positions. (d) Migration in TB and in a 20 mm Ni^2+^ gradient (chemorepellent). Successful migration (i.e., > 1000 swimming cells per field of view, indicated by gray dotted lines) occurred at 9 h when the antibiotic reservoir contained TB alone. Data in (b,d) represent mean ± range from two biological replicates (*n* = 2). TB: tryptone broth. CIP: ciprofloxacin. AMP: ampicillin. GEN: gentamicin. CB: chemotactic buffer. ASP: aspartic acid. AR: antibiotic reservoir. MC: migration channel.

Time‐lapse imaging further illustrated the typical random trajectory of *E. coli* in a 10MIC GEN gradient. Bacteria that entered the antibiotic reservoir returned to the migration channel (being exposed to a constant antibiotic concentration then a gradient, red trajectories in Figure 2c) and vice versa (pink trajectories in Figure 2c, Figure S4 and Video S1). Together, these results demonstrated that *E. coli* randomly migrated within the antibiotic gradients of GEN, CIP and AMP, indicating that *E. coli* does not exhibit chemotactic behavior toward any of the three antibiotics. This finding is consistent with previous studies demonstrating that antibiotics are not chemoeffectors for *E. coli* [21]. The different behaviors of these two species in antibiotic gradients may be attributed to their use of different mechanisms for chemotaxis: *E. coli* uses swimming motility, whereas *P. aeruginosa* uses twitching motility [22].

Since *E. coli* randomly migrate in antibiotic gradients, this stochastic process suggested that the number of cells migrated into the antibiotic reservoir would scale with the starting cell density in the bacterial reservoir. To maintain bacterial metabolic activities and observe their long‐term response in antibiotic gradients, CB buffer was replaced with TB medium. In contrast to CB buffer, TB medium supports rapid cell growth. Therefore, we first characterized *E. coli*’s migration in TB medium without an antibiotic gradient (Figure 2d). During the first 8 h, only a few motile bacteria were present at the observation window near the antibiotic reservoir, and the cell number increased to over 1000 cells/FOV by 9 h. Such an exponential increase corresponded well with cell growth (Figure S5), demonstrating that the number of cells reaching the antibiotic reservoir scaled with the initial population, consistent with bacteria's stochastic random migration. By 10 h, some cells attached to the surface and grew into small colonies (as indicated in Figure S6), and the bacterial density became too high to allow reliable tracking of individual cells. Thus, we defined the threshold for successful migration as the number of motile cells within the observation window, exceeding 1000 cells/FOV at specific time points, with cell numbers continuously increasing over time. Failed migration was defined as bacteria either reaching more than 1000 cells/FOV at specific time points but subsequently decreasing in number or failing to reach 1000 cells/FOV throughout the entire observation period.

To validate this criterion, we supplemented TB medium with 20 mm Ni^2+^, a known chemorepellent compound for *E. coli* [21], in the source reservoir. Under this condition, hardly any bacteria were observed by 12 h at the observation window, and less than 220 cells/FOV were detected by 18 h (Figure 2d), showing unsuccessful migration as expected.

### 2.3 Bacterial Responses and Resistance Development Under Different Classes of Antibiotic Gradients

Next, we characterized *E. coli* dynamic responses during migration in different antibiotic gradients. For GEN, bacteria demonstrated successful migration in all conditions (Figure 3a) by 9 h, even in the gradient of 100MIC GEN. In contrast, successful migration was only detected at low CIP gradients (0.1 and 1MIC) (Figure 3b) and low AMP gradients (0.1, 1 and 10MIC) (Figure 3c).

**FIGURE 3:**
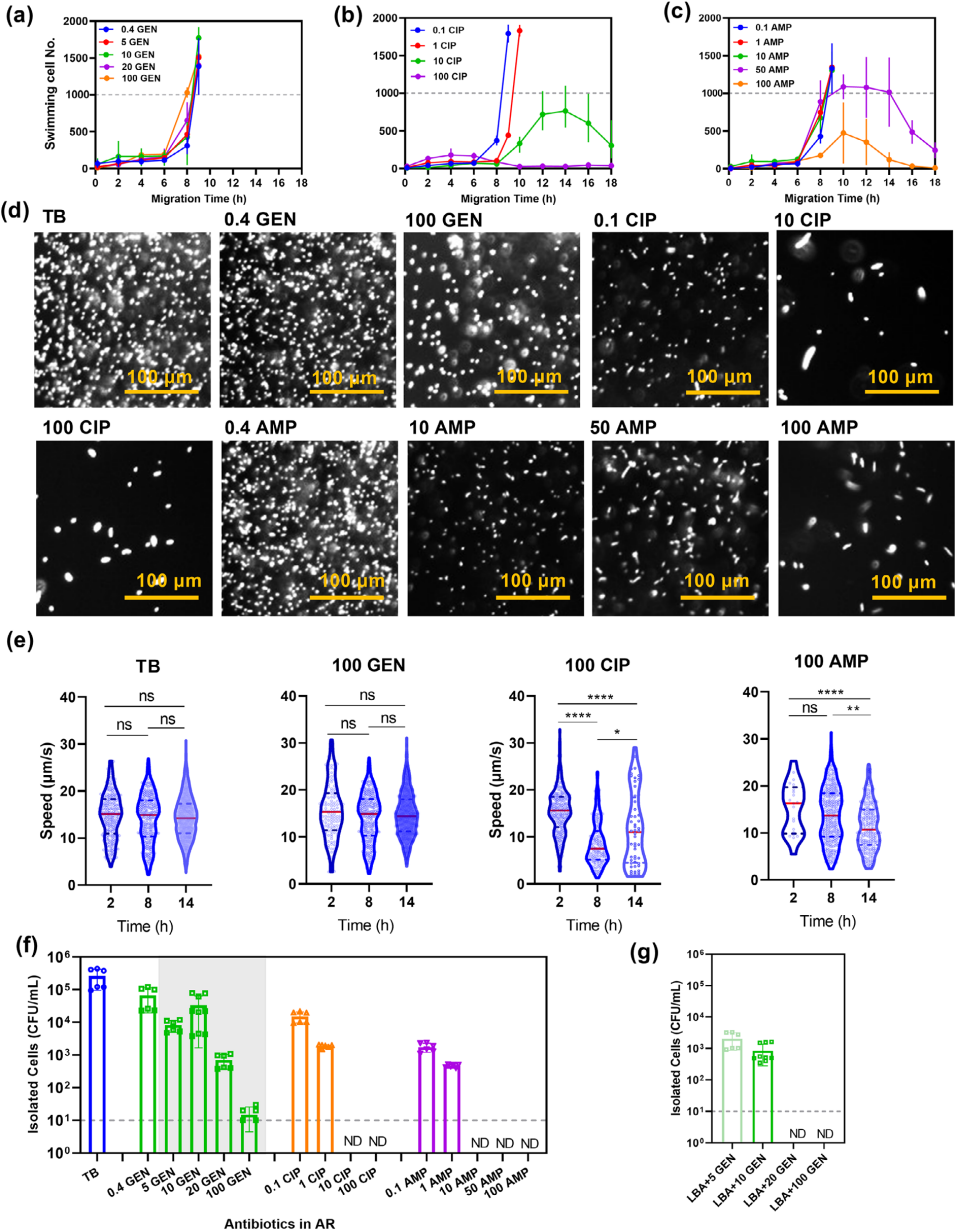
Bacterial migration and subsequent characterization. (a–c) Number of bacteria reaching the observation window over time under gradients of GEN, CIP, and AMP. Numbers preceding antibiotic names indicate fold‐MIC. Data represent mean ± range (*n* = 2 for all experiments except the 10MIC gradient condition, for which *n* = 3). (d) Fluorescence images of bacteria in the migration channel at 10 h. Scale bar: 100 µm. (e) Mean swimming speeds under each condition. In the violin plots, dashed lines represent the first and third quartiles, the solid red line indicates the median, and individual cells (dots). (f) Number of colonies formed on LB agar from retrieved cells (total sample volume = 1 mL). (g) Number of colonies formed on LB agar containing the same GEN concentration as the reservoir (indicated by the shade area in Figure 3f). Gray dotted lines indicate the limit of detection. TB: tryptone broth. GEN: gentamicin. CIP: ciprofloxacin. AMP: ampicillin. ND: not detected. AR: antibiotic reservoir. LBA: LB agar. Statistics: two‐tailed Mann‐Whitney U test (two‐group) and Kruskal–Wallis test with Dunn's post hoc correction (multi‐group). ns: not significant. ^*^ *p* < 0.05, ^**^ *p* < 0.01, ^****^ *p* < 0.0001.

Interestingly, while *E. coli* morphology remained unchanged in successful migrations, many cells became elongated under the conditions of unsuccessful migration, including high CIP gradients (i.e., 10 and 100MIC) and high AMP gradients (i.e., 50 and 100MIC) (Figure 3d and Figure S7). These unsuccessful migrations resulted in fewer bacteria present in the migration channel at the 18‐h endpoint (Figure S8). In addition, no significant changes in bacterial swimming speeds were observed during successful migrations (e.g., in TB medium only or under all GEN gradients) (Figure 3e). In contrast, bacterial swimming speed decreased substantially over time during unsuccessful migrations, particularly under high gradient conditions, for example, after 8 h in the 100MIC CIP gradient and after 14 h under 100MIC AMP gradient (Figure 3e). These decreases correlated well with the observed failure of bacterial migration, indicating that cell elongation reduces their motility. This may explain why *E. coli* achieved successful migration only in the GEN gradient. Morphological changes are a well‐known bacterial response to antibiotic stress. For example, *E. coli* can activate the SOS response upon exposure to CIP and AMP, which often leads to cell elongation, a phenomenon termed filamentation [23, 24, 25]. Bacterial filamentation is an important adaptive strategy that contributes to survival under antibiotic stress and has been implicated in the development of antibiotic resistance [26]. However, this filamentation also decreases bacterial swimming motility, thereby reducing the ability of cells to explore the antibiotic gradient. GEN is a broad‐spectrum bactericidal aminoglycoside [27, 28] and does not affect bacterial morphology or motility [24]. Therefore, the mechanism of antibiotic inhibition is a vital factor influencing bacterial migration in antibiotic gradients.

Interestingly, many bacteria that migrated into the antibiotic reservoir remained mobile even at the highest antibiotic concentrations (Figure S9 and Videos S2–S4). This active movement indicates that these bacteria retain a certain level of viability [9, 29]. To further characterise their metabolic activity and resistance profiles, all cells that migrated into the antibiotic reservoir were isolated off‐chip and cultured on LB agar plates. By sequential introducing air bubbles followed by a PBS wash through the inlet, more than 93% of the bacteria were successfully retrieved from the chip (Figure S10). Additionally, dense biofilms were effectively removed from the reservoirs.

Colonies formed from the migrated cells (i.e., cells that had entered the antibiotic reservoir) under all GEN gradients (Figure 3f), with more than 10^4^ CFU/mL recovered under the 0.4MIC GEN gradient and 15 CFU/mL under the 100MIC GEN gradient. However, bacterial colonies were only detected in the CIP and AMP groups at gradients < 1MIC (Figure 3f). No colonies were recovered from the antibiotic reservoir under CIP or AMP gradients greater than 1xMIC, and under these conditions, bacteria failed to achieve successful migration. This suggested that these migrated cells were non‐growing despite exhibiting visible motility (Videos S3 and S4). This phenomenon may contribute to the induction of SOS response, which can lead to DNA damage and thereby inhibit cell division [15]. These results show a strong correlation between successful migration and the presence of metabolic‐active and culturable cells in the antibiotic reservoir.

Since the ancestral strain was susceptible to all antibiotics tested, we asked whether viable cells in the antibiotic reservoir could grow there, as growth at lethal concentrations is a clear indication of resistance. Because all GEN gradients showed successful migrations at reservoir concentrations > 1MIC, cells collected from these conditions were plated on LB agar containing the same GEN concentration as in the reservoir. No colonies formed from cells exposed to 20MIC GEN and 100MIC GEN gradients (Figure 3d), whereas substantial colony formation (∼ 1000 CFU/mL) was observed for cells collected from the 5MIC GEN and 10MIC GEN gradients (Figure 3d), suggesting that these cells had developed tolerance or resistance to the antibiotic [10]. It is worth noting that bacterial filamentation was observed in the sub‐MIC regions after 18 h under the 10MIC CIP and 10MIC AMP gradient conditions but not under the 10MIC GEN gradient condition (Figure S11a). To investigate whether bacteria develop resistance in the sub‐MIC or low antibiotic regions, we retrieved bacteria from the migration channel and bacterial reservoir after 18 h under these gradient conditions (Figure S11b). Numerous colonies were observed on LB agar in all groups. However, colonies were detected on antibiotic‐containing agar only among bacteria collected under the 10MIC GEN gradient (Figure S11c). These results indicate that cells under sub‐MIC or low concentrations of CIP and AMP did not develop stable resistance within 18 h. Under these conditions, filamentation was triggered to resist antibiotic stress, but it also reduced cell motility. In contrast, the resistant cells collected from the migration channel under the 10MIC GEN gradient were likely derived from the random migration of resistant cells from the antibiotic reservoir into the migration channel, as indicated by the high density of bacteria in the migration channel (Figure S11a). Together, these findings illustrate that bacterial adaptation and resistance to antibiotics are multifaceted processes, in which successful migration across the antibiotic landscape is a prerequisite.

### 2.4 The Critical Role of Migration Duration in Resistance Development Under Gradient Exposure

Since antibiotic treatment duration can determine bacterial survival [30, 31], we next examined how migration duration affects bacterial adaptation in antibiotic gradients. We performed two types of time‐course experiments using 10MIC GEN gradients (Figure 4): (a) Experiments with the same migration and exposure durations, in which cells migrated in the gradients for 1, 5, 10, or 15 h before being retrieved off the chip; (b) Experiments with different migration durations with a fixed total exposure of 18 h. For migration durations shorter than 18 h, after closing the migration channel, cells remained in the antibiotic reservoir before retrieval off chip.

**FIGURE 4:**
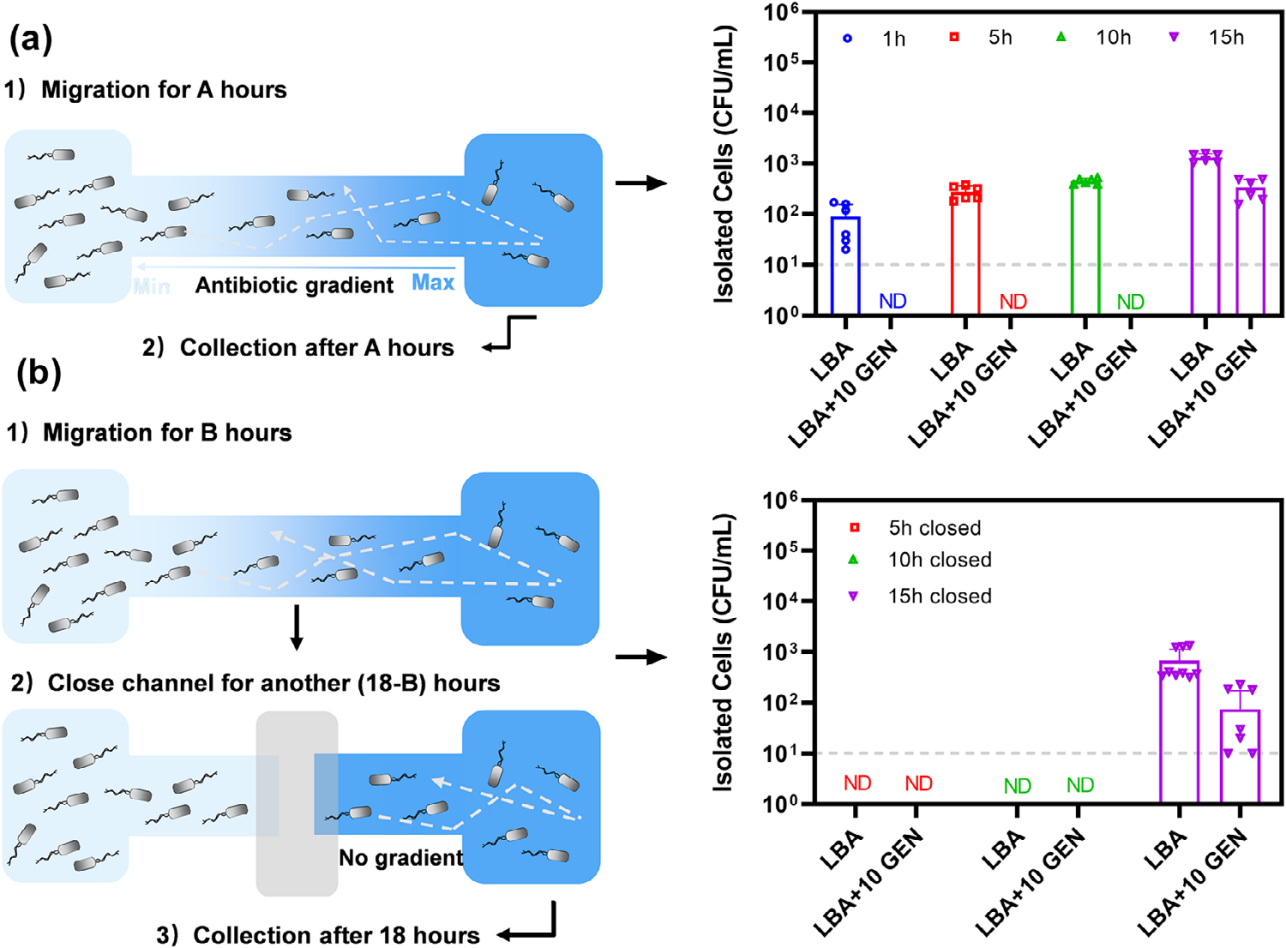
Effects of migration duration on migration and adaptation. (a) Number of colonies formed on agar plates from cells collected after varying migration durations in a 10MIC GEN gradient. (b) Colony formation from cells retained in the reservoir until 18 h after initial exposure to a 10MIC GEN gradient for 5, 10, or 15 h. Gray dotted lines indicate the detection limit. LBA: LB agar.

Because bacterial migration in antibiotic gradients is stochastic, the number of bacteria reaching the antibiotic reservoir increased with migration time (Figure S12). For cells retrieved directly at different migration durations (i.e., type‐(a) experiments), colonies formed on LB agar for all conditions. However, only cells collected after 15‐h migration formed colonies on LB agar containing GEN (Figure 4a), indicating the development of resistance. Additionally, this resistant sub‐population accounted for 27.9% of the total viable population (i.e., the total colonies formed on LB agar). These results show that a sufficient migration period across the antibiotic landscape is essential for bacteria to develop resistance to lethal antibiotic stress.

Based on this finding, we next asked whether extending migration time could enable bacteria adaptation to steeper antibiotic gradients, for example, 20MIC GEN gradient, under which an 18‐h migration yielded no colonies formation (Figure 3g). Interestingly, when the migration duration was extended to 48 h under 20MIC GEN gradients in TB or LB medium, a single colony (denoted as L‐20G‐1) was detected on the antibiotic agar, showing that prolonged migration can promote the emergence of resistant cells under steeper gradients, albeit at a very low frequency.

However, for cells first exposed to gradients and subsequently to uniform antibiotic concentrations in the reservoirs (i.e., type‐(b) experiments), no colonies formed from the collected cells when the migration channel was closed after 5 or 10 h (Figure 4b). Colonies only appeared on LB agar with or without antibiotics when the migration channel was closed after 15 h. These results indicate that the development of resistance or tolerance is primarily associated with sufficient exposure time to the antibiotic gradient rather than exposure to fixed GEN concentrations. When GEN gradients were eliminated in both microfluidic and bulk experiments, no growing cells were recovered on antibiotic agar (Figure S13), further confirming that antibiotic gradients, rather than fixed concentrations, are essential for resistance development.

### 2.5 Key Factors Affecting Successful Migration and Resistance Development

#### 2.5.1 Effect of Initial Population Loading Density

Since bacterial migration is a stochastic process, this suggests that the number of cells migrating into the antibiotic reservoir would scale with the starting cell densities in the bacterial reservoir. Initial trials showed that high loading densities (> 10^8^ cells/mL) in the bacterial reservoir did not allow reliable in situ observation due to the large number of randomly migrating cells. Thus, a loading density of 10^7^ cells/mL was used for all tests unless otherwise noted. With a density of 10^7^ cells/mL in TB medium, the number of migrating cells increased exponentially around 9 h in all successful migrations (Figures 2d and 3a–c), suggesting the existence of a threshold cell density.

To evaluate this, we examined loading densities of 10^6^ cells/mL and 10^5^ cells/mL and evaluated cell migration under 10MIC GEN gradients. Successful migrations occurred for both densities, albeit with longer durations, specifically at 16 h for 10^6^ cells/mL and at 25 h for 10^5^ cells/mL (Figure 5a). Growth curves showed that similar OD values were reached at these time points for the three loading densities (Figure S14).

**FIGURE 5:**
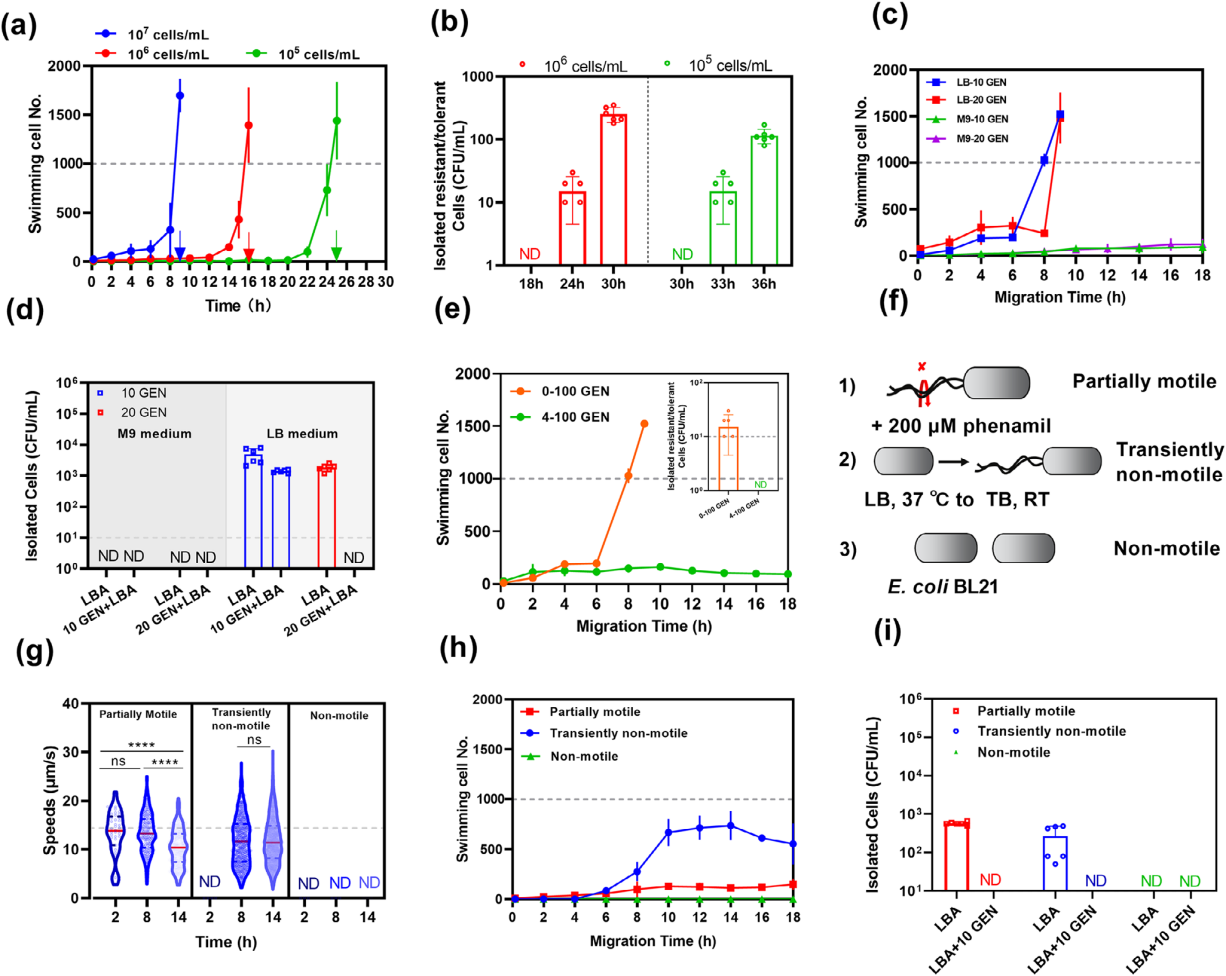
Key factors influencing migration and adaptation. (a) Migration curves (i.e., number of bacteria reaching the antibiotic reservoir over time) under different initial loading densities in a 10MIC GEN gradient. (b) Colony numbers on LB agar containing 10MIC GEN across loading densities and migration durations. (c) Migration curves under 10MIC and 20MIC GEN gradients in LB or M9 medium. (d) Conoly formation with or without antibiotics from cells collected under different media. (e) Migration curves under 100MIC GEN gradients for untreated (0‐100 GEN) or pre‐treated (4‐100 GEN) cells (i.e., cells were treated with 4xMIC GEN in the bacterial reservoir). Inset: colony numbers on LB agar containing 10MIC GEN. (f) Three types of motility‐compromised bacteria. (g) Swimming speeds of motility‐compromised bacteria. ND: No swimming bacteria detected. The gray dashed line indicates the median swimming speeds without treatment. (h) Migration curves and (i) colony formation under 10MIC GEN gradients for each motility‐compromised condition. Data represent mean ± range from two biological replicates (*n* = 2). TB: tryptone broth. LB: lysogeny broth. M9: M9 minimal medium. LBA: LB agar. GEN: gentamicin. ND: not detected. Statistics: Mann‐Whitney U test (two‐group) and Kruskal–Wallis with Dunn's post hoc test. ns: not significant; ^****^ *p* < 0.0001).

Similarly, colony formation on antibiotic agar was only observed from cells collected after sufficient migration durations, namely, after 24 h for a loading density of 10^6^ cells/mL and after 33 h for 10^5^ cells/mL (Figure 5b). Interestingly, across all tested densities, the time interval between the point of successful migration and the emergence of resistance remained relatively consistent (e.g., between 18 and 24 h for 10^6^ cells/mL), at approximately 6–8 h. These results demonstrate that resistance evolution occurs only when a sufficient number of migrated bacteria migrate through the antibiotic gradients. Consequently, achieving a critical population size is essential for the successful establishment of a resistant population.

#### 2.5.2 Effect of Cell Growth

To corroborate these findings, we further evaluated common factors affecting cell growth, namely nutrient availability and antibiotic inhibition. Nutrients often vary in real‐world settings; thus, we compared nutrient‐rich (LB) and nutrient‐poor (M9) media with TB medium (Table S1). As expected, *E. coli* grew faster in LB medium than in TB medium and showed no visible growth in M9 medium within 22 h (Figure S15). In LB medium, successful migration and colony formation from collected cells were observed (Figure 5c,d). However, no successful migrations and no colonies were observed under any GEN gradient with M9 medium (Figure 5c,d). These observations suggest that a nutrient‐rich environment supports exponential growth of bacteria and active motility, which may explain the low levels of resistance observed previously in M9 medium [14].

Similarly, when initial cell growth was inhibited by antibiotics, for example, by pre‐treating *E. coli* with 4 × MIC GEN in TB medium, bacterial growth was completely suppressed (Figure S16), and no successful migration was observed (Figure 5e). Consequently, no colonies were detected under growth‐inhibited conditions (inset figure in Figure 5e). These findings suggest that suppressing cell growth can delay the emergence of resistance.

#### 2.5.3 Effect of Bacterial Motility

Within a fixed antibiotic gradient, bacterial motility can modulate how bacterial cells explore the gradient. The compromised mobility of *E. coli* caused by CIP and AMP resulted in unsuccessful migration and the absence of resistant cells (Figure 3 and Figure S11). To understand the effects of cell motility, we further examined *E. coli* with compromised motilities (Figure 5f): (1) Partially motile cells, achieved by treating the ancestral *E. coli* strain with 200 µm phenamil, which inhibited flagellar rotation [32]. This treatment resulted in drastically reduced motility while remaining their viability (Figure 5g and Figure S17a–c). (2) Transiently non‐motile. This was achieved via culturing motile bacteria under optimal growth conditions (e.g., LB medium at 37°C), which temporarily inhibited motility. Motility was restored by altering the culture conditions (e.g., TB medium at room temperature; Figure 5g) [33]. (3) A non‐motile *E. coli* strain BL21 [34], which was used as a negative control (Figure 5g and Figure S17d,e).

None of the motility‐compromised cells achieved successful migrations under 10MIC GEN gradients, and none yielded resistant cells (Figure 5h,i). These observations underscore the determinant role of sufficiently active motility, which enables bacteria to explore antibiotic gradient landscapes for adaptation. Our results provide insights to the findings in previous studies, where reduced resistance development was observed under suboptimal culture conditions [9] or the use of motile‐deficient strains [14].

### 2.6 Verification of Resistance Profiles and Gene Mutations

To confirm that resistance arose from exposure to antibiotic gradients rather than from pre‐existing resistance, heteroresistance assays were performed on the ancestral strain. Heteroresistance refers to a phenomenon in which subpopulations of isogenic bacteria exhibit a range of susceptibilities to a given antibiotic [35]. If no colonies appeared inside the inhibition zone, the strain was considered non‐heteroresistant. A clear inhibition zone (diameter, 24 ± 2 mm; Figure S18) showed onto a 10 µg GEN disk, indicating GEN susceptibility (CLSI criteria: susceptible if inhibition zone ≥ 18 mm) and the absence of heteroresistance [35]. Tolerance is the ability of microorganisms to survive transient exposure to high concentrations of an antibiotic without a change in the MIC value [30]. An additional tolerance disk test was conducted, in which no colonies appeared after antibiotic removal and a further 24‐h incubation following glucose addition, confirming a lack of tolerance [36] (Figure S18). These results confirm the absence of pre‐existing resistance and tolerance in the ancestral strain.

To characterise the resistance profiles of viable cells isolated from the antibiotic reservoir, single colonies on antibiotic agar were randomly picked and their MICs were determined. No resistant strains emerged in 5MIC GEN gradients among 11 randomly picked strains (Figure S19); some displayed either identical or 2‐fold increased MIC values compared to the ancestral strain, consistent with previously defined characteristics of tolerant or persistent cells (unchanged MIC) [37] or partially resistant cells (with slightly increased MIC) [38]. In contrast, a mixed population was observed in 10MIC GEN gradients (Figure 6a), consisting of susceptible, intermediate, and resistant subpopulations based on CLSI breakpoints for GEN (MIC ≤ 2 mg/L for susceptible, ∼ 4 mg/L for intermediate, and ≥ 8 mg/L for resistant) [20]. This heterogeneous response suggests that *E. coli* can adopt diverse adaptive pathways when exposed to 10MIC GEN gradients—a phenomenon normally associated with an increased risk of treatment failure and recurrent infections [5, 30, 38].

**FIGURE 6:**
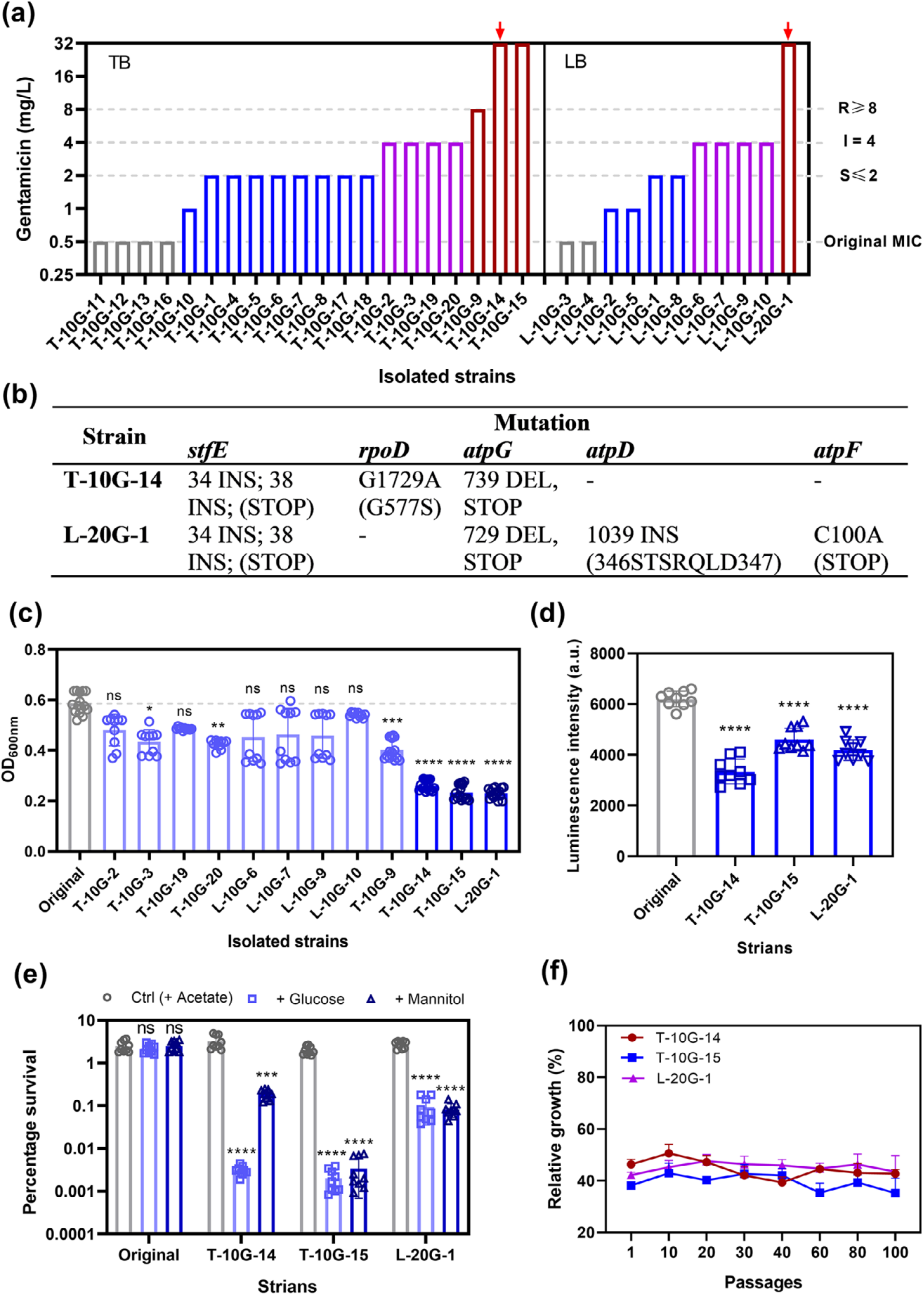
Verification of antibiotic resistance. (a) MICs of randomly selected colonies on antibiotic agar. Gray dotted lines indicate the original MIC value and the MIC breakpoints for susceptible, intermediate, and resistant phenotypes, respectively. All strains except L‐20G‐1 were isolated from 10MIC GEN gradients after 18 h. L‐20G‐1 was isolated after 48 h under 20MIC GEN gradients. (b) Mutations identified in resistant strains T‐10G‐14 and L‐20G‐1. DEL: deletion; INS: insertion; STOP: gain of a stop codon resulting in the premature termination of transcription. The site indicates the nucleotide position within the corresponding genes. (c) Growth of intermediate and resistant strains compared to the ancestral strain. (d) Intracellular ATP levels in the resistant strains and the ancestral strain. (e) Survival percentage in the resistant strains and the ancestral strain supplemented with different carbon sources. (f) Relative growth after 100 passages. MIC: minimum inhibitory concentration. S: susceptible. I: intermediate. R: resistant. Statistics: Kruskal–Wallis test with Dunn's post hoc correction for Figure 6c, One‐Way ANOVA with Dunnett's post hoc correction for Figure 6d, Two‐Way ANOVA with Dunnett's post hoc correction for Figure 6e. ns: not significant; ^*^ *p* < 0.05, ^**^ *p* < 0.01, ^***^ *p* < 0.001, ^****^ *p* < 0.0001.

PCR testing of non‐susceptible strains (MIC ≥ 4 mg/L) detected no known gentamicin‐resistance genes, including *aad B*, *aac(6* ***′*** *)‐Ie‐aph(2* ***′*** *)‐Ia*, *aac(3)‐VI*, *aacC2*, and *aac(3)‐IV* (Figure S20). Accordingly, whole genome resequencing was performed to identify genetic variants between the ancestral strain and the gentamicin‐resistant strains T‐10G‐14 and L‐20G‐1, both of which exhibited increased MIC values of 32 mg/L (Figure 6a).

Mutations in *stfE*, *rpoD, atpD, atpG*, and *atpF* were identified at multiple sites in both strains (Figure 6b and Table S2). Among these, no evidence currently links mutations in *stfE* or *rpoD* to gentamicin resistance. A single‐nucleotide deletion at site 739 and a four‐nucleotide deletion at site 729 in the *atpG* gene were detected in strains T‐10G‐14 and L‐20G‐1, respectively, both predicted to result in premature termination of transcription. In addition, an insertion in *atpD* and a deletion in *atpF* were identified in strain L‐20G‐1. The former can result in an amino acid insertion (346STSRQLD347) in the *AtpD* protein, while the latter can terminate *atpF* transcription in advance.

The genes *atpG*, *atpD*, and *atpF* encode subunits of the F_1_F_0_H^+^‐ATPase (ATPase), which catalyses ATP synthesis. This enzyme comprises eight subunits (i.e., *atpB, atpE, atpF, atpH, atpA, atpG, atpC, and atpD*), all of which are expressed from a single operon under a common promoter [39]. Deletion of *atpG* and *atpF* has been shown to increase gentamicin resistance in *E. coli* [40]. Consistently, genes encoding ATP synthase subunits, including *atpF*, *atpG*, and *atpD*, were among the most strongly downregulated following gentamicin treatment [41]. Loss of these subunits may reduce gentamicin uptake, thereby increasing resistance [42]. However, this resistance mechanism appeared specific to GEN and did not extend to other antibiotic classes (e.g., CIP and AMP; Figure S21).

Next, the growth rates of these mutant strains were characterized, revealing a trade‐off between resistance and growth (Figure 6c). All strains with MIC values ≥ 8 mg/L (classified as resistant) exhibited reduced growth rates compared to the ancestral strain. Among them, the resistant strains (T‐10G‐14, T‐10G‐15, and L‐20G‐1), which displayed higher MIC values, showed more pronounced reductions in growth, likely due to deletions in ATP‐related genes.

To further validate ATP depletion in the resistant strains, intracellular ATP levels were quantified using a luciferase/luciferin‐based bioluminescence assay. The luminescence intensities of the gentamicin‐resistant strains T‐10G‐14, T‐10G‐15, and L‐20G‐1 were markedly lower than those of the ancestral strain (Figure 6d), indicating reduced intracellular ATP production in the resistant strains. ATP depletion is expected to further reduce the proton motive force (PMF) [5]. Previous studies have demonstrated that metabolites entering upper glycolysis, such as glucose and mannitol, enhance the proton motive force, thereby facilitating aminoglycoside uptake [43]. As indicted in Figure 6e, supplementation with glucose or mannitol significantly reduced the survival percentage of the resistant strains following gentamicin treatment compared with acetate (which does not enhance the proton motive force), whereas no such effect was observed in the ancestral strain. These findings suggest that the resistant strains have a depletion of ATP production and diminished proton motive force, resulting in reduced gentamicin uptake and consequently contributing to antibiotic resistance.

However, unlike plasmid‐mediated resistance [44], these reduced growths were not reversible, nor were they partially compensated by serial passaging (Figure 6f). Moreover, MIC values remained unchanged after 100 serial passages, indicating that the acquired resistance was highly stable. Importantly, reduced growth rates may promote tolerance or dormancy during antibiotic treatments, often necessitating prolonged therapy and increasing the risk of treatment failure [30].

### 2.7 Widespread Resistance Development in Other Motile Strains Under Gradients

To investigate whether resistance development under antibiotic gradients is a more general phenomenon among motile bacterial species, two additional strains were tested, including *E. coli* MG1655 (lacking any antibiotic resistance markers) and *S. marcescens*. Both strains exhibit active motility. We observed large populations of motile bacteria migrating through the observation window at approximately 9 h for *S. marcescens* under the 10MIC GEN gradient (Video S5). A similar migration pattern was also observed for *E. coli* MG1655.

After 18 h of migration, bacteria were retrieved from the antibiotic reservoir, and colonies were observed on gentamicin‐containing agar for both strains (Figure 7a), indicating the emergence of gentamicin resistance. Subsequently, 12 randomly selected colonies from each strain were isolated, cultured, and their MIC values were measured to further assess resistance development. A heterogeneous population with different gentamicin susceptibilities was observed in both strains. Importantly, gentamicin‐resistant isolates were identified in both bacterial species. Together, these results further support our conclusion that motile bacteria can rapidly develop resistance under antibiotic gradients when they are able to sufficiently explore the gradient landscape.

**FIGURE 7:**
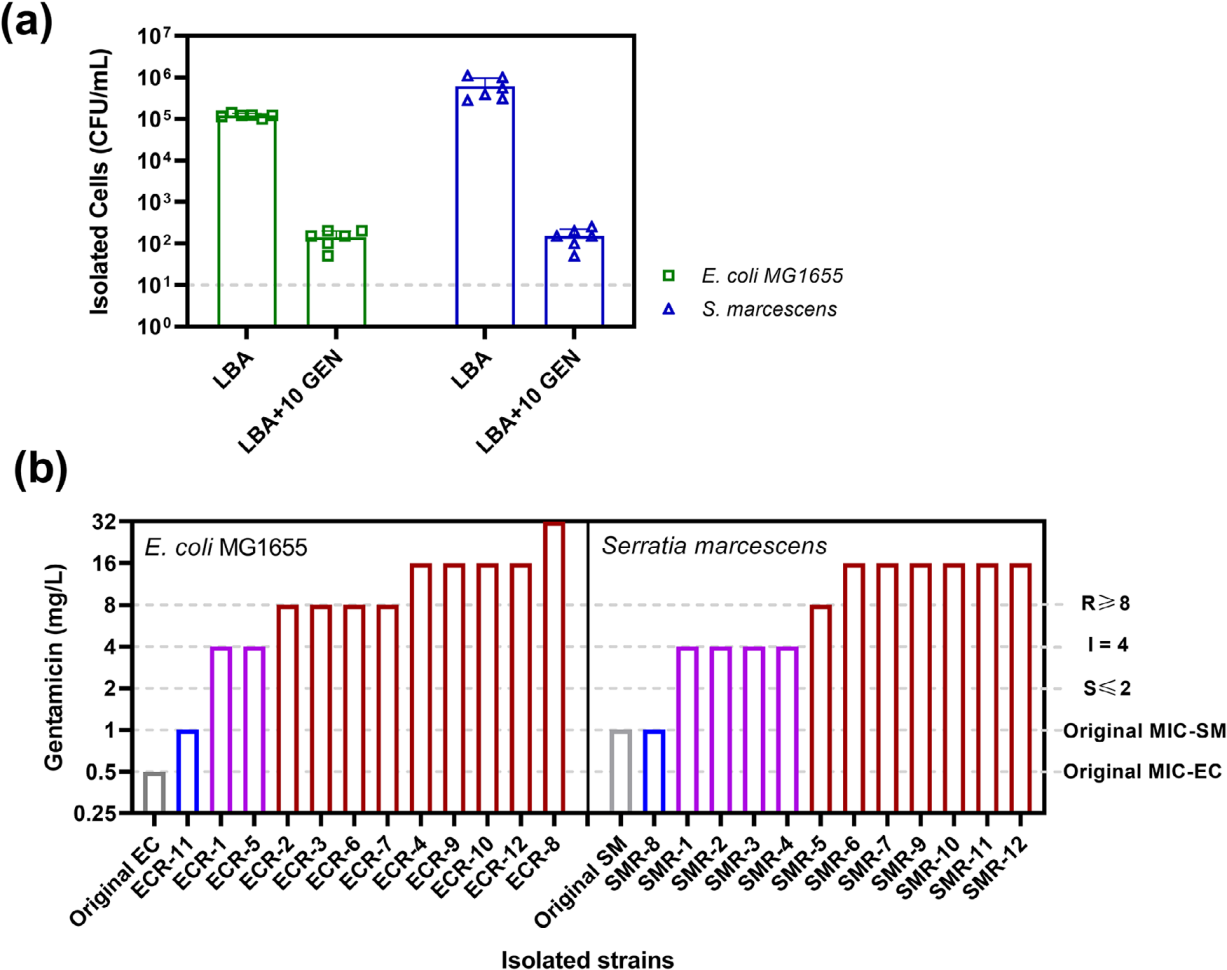
Resistance Development of Other Motile Bacterial Strains Under 10MIC GEN Gradients. (a) Numbers of bacteria isolated from the antibiotic reservoir (AR) for *E. coli* MG1655 and *S. marcescens* under 10MIC GEN gradient conditions. Both strains developed gentamicin resistance after exposure to the antibiotic gradient. (b) 12 randomly selected single colonies isolated from the AR under 10MIC GEN gradient conditions were cultured, and their MIC values were subsequently determined for both strains. Gentamicin‐resistant strains were identified in both strains. Gray dashed lines indicate the original MIC values and the MIC breakpoints for susceptible, intermediate, and resistant phenotypes, respectively.

## 3 Discussions

Spatially heterogeneous antibiotic concentrations are ubiquitously present in natural habitats, often associated with increased antibiotic resistance and treatment failures [8, 45]. Theoretically, bacterial evolution in heterogeneous antibiotic landscapes has been described using quantitative staircase models, in which bacterial populations evolve through stochastic processes of proliferation, migration, mutation, and death [13, 16]. Numerical results suggest that cell growth [13] and bacterial motility [16] can govern bacterial adaptation. However, the assumptions on which these predications were based are challenging to meet in traditional experimental settings. Indeed, discrepancies were found in many studies, which were attributed to differences in species, bacterial growth conditions, and experimental settings [9, 10, 11, 12, 13, 14, 15]. Therefore, well‐controlled experimental conditions are critically important for investigating the evolution of antibiotic resistance under heterogeneous antibiotic gradients.

Gel‐based or flow‐based microfluidics are common formats used to create concentration gradients [9, 46]. However, these methods are prone to flow disturbances, and actively retrieving cells of interest off‐chip is challenging due to the enclosed nature of the devices. In contrast, our microfluidic platform not only generates long‐term, well‐controlled antibiotic gradients but also enables user‐defined temporal control of bacterial exposure, either in gradient or homogeneous conditions, before isolating the migrated subpopulation off‐chip for further resistance profiling. This capability allows real‐time characterization of dynamic single‐cell responses (including motility, morphology, and swimming trajectory) and direct correlation with the emergence of adaption and resistance within a population.

Using a susceptible *E. coli* strain, we examined the roles of key factors, including cell motility, migration time, and growth in the evolution of antibiotic resistance under gradients. Confounding results on these factors commonly arise in growth‐based assays reported previously [9, 10]. With the powerful microfluidic platform, we were able to control and characterise each factor independently and correlate their dynamic contributions to resistance evolution. Our results showed that *E. coli* migration in GEN, CIP, and AMP gradients was driven by unbiased and random swimming, suggesting that the process is largely stochastic (Figure 2a–c). Bacterial adaptation in these gradients is accelerated by the population's ability to migrate across the heterogeneous landscape for an extended duration. In this process, bacterial motility plays a dominant role, as sufficient motility enabling spatial navigation across gradients is a prerequisite for resistance development. Inhibiting cell motility was effective in limiting bacterial adaptation, preventing the formation of tolerance and resistance (Figure 5g–i). Thus, a combination of antibiotics with drugs that compromise bacterial motility could offer a promising strategy to impede the evolution of antibiotic resistance.

An important finding is that a minimum time of migration within gradients is required to establish resistance mutations within a growing population, although shorter durations can also accelerate bacterial adaptation, but result in tolerant cells (Figure 4). This is reasonable as resistance mutations are rare events, thus from a statistical perspective, a critical number of cells are needed. In addition, although migration to lower concentrations provides temporary “sanctuaries” from antibiotic stress, repeated exposures promote iterative tolerant mutations which can accelerate resistance emergence [47]. Whole‐genome resequencing revealed mechanisms of resistance adaptation, and a trade‐off between antibiotic resistance and growth was observed.

Our results also showed that active growth is important for generating a critical mass required for the evolution of antibiotic resistance. For example, nutrient‐poor M9 medium, which did not support sufficient growth during the observation period, effectively reduced the occurrence of tolerant or resistant cells (Figure 5d). This finding is in good agreement with Hol's work [9], suggesting that a critical population density is needed to successfully establish a viable resistant population. However, the effects of bacterial motility were masked by cell growth in most of previous studies. Our studies demonstrated the prominent role of motility—compromised motility can effectively reduce resistance evolution even in the presence of a high cell population. When *E. coli* became elongated with reduced motility under high concentration CIP and AMP gradients (>10 MIC), no resistant cells were detected after 18 h despite of increasingly growing cell density in the bacterial reservoir (Figure 3). These findings highlighted targeting bacterial motility can be an effective strategy for reducing the development of resistance in motile bacteria in real‐world applications.

However, our current study primarily focuses on the resistance development of swimming motile bacteria under antibiotic gradient conditions. Apart from swimming motility, bacteria also exhibit other forms of motility, such as swarming, twitching, gliding, and sliding [22]. The surface of the microfluidic chip can be modified to support these modes of motility, thereby extending the applicability of the platform. In addition, even non‐motile bacteria, such as *Staphylococcus aureus*, can employ phenol‐soluble modulin‐mediated movement to achieve rapid colonization of wet surfaces [48]. Future work will extend this platform to investigate antibiotic resistance development in non‐motile bacteria by dynamically modulating antibiotic gradients in both space and time, and by integrating the detection of SOS responses and other metabolic pathways to elucidate the underlying resistance mechanisms. For example, the platform could be coupled with an online microfluidic mass spectrometry system [26] to enable real‐time metabolic profiling.

## 4 Conclusions

In conclusion, this work demonstrates that microfluidics provides a powerful tool for dissecting the critical determinants underlying antibiotic resistance evolution in heterogeneous antibiotic landscapes that are ubiquitous in real‐world environments. We show that antibiotic gradients can accelerate the evolution of antibiotic resistance. In this process, bacterial motility plays a dominant role, followed by cell growth. These findings suggest that reducing bacterial motility can offer a simple and effective strategy to impede the evolution of resistance in motile bacteria, thereby opening a new avenue to tackle antibiotic resistance and extend the useful lifespan of existing antibiotics. In this context, the microfluidic platforms, as demonstrated in this work, hold great promise for the rapid evaluation of innovative therapeutic strategies.

## 5 Experimental Section

### 5.1 Microfluidic Chips Fabrication

A three‐dimensional (3D) microfluidic platform was fabricated as described previously [18]. In brief, two metal needles were positioned in the designated slots on a 3D printed microfluidic mould before pouring a mixture of polydimethylsiloxane (PDMS) oligomer and curing agent (20:1, wt/wt; Sylgard 184, Dow Corning). After curing, the needles were removed to form the valve channels, and the PDMS layer was peeled off from the mould and bonded to coverslips using oxygen plasma. The device allows programmable solution delivery, maintains stable gradients for over 20 h, and enables retrieval of bacteria off‐chip, as illustrated in Figure S1.

### 5.2 Bacterial Strain and Culture, Antibiotics Solution Preparation

A kanamycin‐resistant (*aph(3')‐Ia* gene, which encodes aminoglycoside O‐phosphotransferase and confers resistance to kanamycin but not gentamicin) and RFP‐expressing *E. coli* strain (MG1655:: P_LacUV5_‐mRFP) was used as the ancestral strain. The strain was cultured overnight in tryptone broth (TB) supplemented with 50 mg/L kanamycin at 30°C and 200 rpm. A 100 µL aliquot of the overnight bacterial suspension was inoculated into 3 mL of fresh TB medium and grown to the exponential phase (OD_600nm_ = 0.5). Subsequently, the bacteria were harvested by centrifugation at 2000 rpm for 3 min. The bacterial pellet was resuspended in fresh TB and diluted tenfold with fresh TB, yielding a motile bacterial solution for subsequent experiments. Two additional strains, including non‐fluorescent *E. coli* MG1655 and a *S. marcescens* strain, were cultured in pure TB medium and prepared in the same way. Unless otherwise noted, all tests were performed under this treatment to ensure consistent motility and density.

All antibiotics used in this study were purchased from Sigma–Aldrich, except ciprofloxacin from Honeywell Fluka. All antibiotics were prepared as stock solutions with concentrations ≥ 10 mg/L and stored at ‐80°C until use.

### 5.3 *E. coli* Migration in Antibiotic Gradients

The chip was primed with 70% ethanol followed by deionized distilled water (dd H_2_O) to remove air bubbles from the channels. It was then treated with 1% Pluronic F‐127 solution for 1 h to minimize cell attachment, followed by washing with ddH_2_O to remove the residual solution. The migration channel was closed via the on‐chip valve, and the bacteria suspension and antibiotic solutions (various concentrations of gentamicin, ampicillin, and ciprofloxacin in TB medium or other medium indicated) were loaded into their respective reservoirs. Both inlet and outlet channels were closed to prevent convection. Time‐lapse images were recorded after opening the migration channel. Unless otherwise noted, the whole observation duration was 18 h. More details are provided in the Supporting Information.

### 5.4 Retrieval of Bacteria Off‐Chip

After the on‐chip assay, bacteria that migrated into the antibiotic reservoir were retrieved off‐chip for further analysis. The collected bacterial suspension was centrifuged at 8000 rpm for 10 min to remove residual antibiotic and resuspended in 1 mL PBS. An aliquot (100 µL) of the bacterial suspension was plated on lysogeny broth (LB) agar plates to quantify total cultivable bacteria. Simultaneously, the same bacterial suspension was plated on LB agar plates containing antibiotics at the same concentration as the reservoir to identify resistant/tolerant bacteria [10]. The plates were incubated at 37°C for 2 days, and colonies on each plate were counted. Single colonies from antibiotic‐containing plates were randomly selected for enrichment and MIC testing to confirm resistance/tolerance. Resistant bacteria were defined according to the breakpoint of MIC values provided by the CLSI [19]. For instance, bacteria were considered resistant to gentamicin when the MIC value was ≥ 8 mg/L. The identified resistant strains were stored at −80°C with 25% glycerol.

### 5.5 Key Factors Involved in the Development of Bacterial Resistance

Bacterial survival under antibiotic stress depends on treatment period and physiological states. Accordingly, key factors, including migration time, nutrient availability, and cell motility, were examined. Three media (M9 minimal medium, TB, and LB; compositions in Table S1) were used to prepare bacterial and antibiotic solutions. Several migration durations were tested. After each migration period, the migration channel was closed to interrupt the gradient. For any migration periods < 18 h, bacteria present in the antibiotic reservoir were retained until a total of 18 h before cells being collected off chip.

Bacteria with three motility levels were prepared:

1. Partially motile, achieved by treating the ancestral strain with 200 µm phenamil methanesulfonate salt (referred to as phenamil hereafter);
2. Transiently non‐motile, achieved by culturing the ancestral strain in LB at 37°C;
3. Non‐motile, using *E. coli* strain BL21.

### 5.6 Whole Genome Sequencing

The ancestral strain and gentamicin‐resistant strains T‐10G‐14 and L‐20G‐1 were cultured in LB to log phase. Genomic DNA was extracted using the PureLink Genomic DNA kit (Life Technologies). Whole genome sequencing was performed on an Illumina NovaSeq 6000 platform (2 × 150 bp paired‐end reads; Eurofins). Base calls with < 99% accuracy were removed. Reads were aligned to the *E. coli* MG1655 (GenBank No. U00096.3) using Sentieon's Haplotype Caller. Variants with coverage < 30× or allele depth < 3× were filtered using Eurofins High Confidence Filter. Only filtered variants were considered as single nucleotide polymorphisms (SNPs) or insertions and deletions (InDels). Mutations presented in the ancestral strain were excluded.

### 5.7 Intercellular ATP Measurement and Survival Percentage

The intracellular ATP concentration of the resistant strains was quantified using the BacTiter‐Glo Microbial Cell Viability Assay kit (Promega, G8230). The survival percentages of bacteria in M9 salts medium supplemented with different carbon sources were determined following a previously reported method [43]. Details are provided in the Supporting Information.

### 5.8 Bacterial Serial Transfer

To evaluate the stability of resistance and growth rates, glycerol stocks of the identified resistant strains were cultured in LB at 37°C overnight, denoted as passage 1. Every 24 h, 2 µL of culture was transferred to 2 mL fresh LB. A total of 100 generations were obtained, assuming 10 generations per day [49]. OD_600nm_ was measured every 10 generations. Net OD values were obtained by subtracting the blank medium control. Growth of the ancestral strain was set to 100%, and relative growth was calculated as:

$$\begin{array}{ccc} & & \mathrm{Relative}\;\mathrm{growth}\;\left(\%\right)\\ & & =\left(\mathrm{net}\;OD_{600\mathrm{nm}}\;\mathrm{of}\;\mathrm{resistance}\;\mathrm{strain}\;/\right.\\ & & \left.\;\mathrm{net}\;OD_{600\mathrm{nm}}\;\mathrm{of}\;\mathrm{original}\;\mathrm{ancestral}\;\mathrm{strain}\right)\times 100\% \end{array}$$

### 5.9 Widespread Resistance Development in Other Strains

To test whether other motile bacterial species can also develop resistance under antibiotic gradients, two additional strains were evaluated under the 10MIC GEN gradient, including *E. coli* strain MG1655 (lacking any antibiotic resistance markers) and *Serratia marcescens*. If bacteria collected from the antibiotic reservoir formed colonies on antibiotic‐containing agar, 12 colonies were randomly selected for subsequent determination of their MIC values to assess the emergence of resistance.

### 5.10 Image Acquisition and Analysis

The microfluidic chip was mounted on an automated motorized stage (SCAN IM 120 × 80–2 mm, Marzhauser Wetlar GmbH & Co. KG, Germany) of an IX71 epifluorescence inverted microscope (Olympus Inc., UK) with an Andor iXon Ultra 897 CCD camera. Imaging was controlled via Micro‐Manager. Unless otherwise specified, fluorescence images of the RFP‐labeled *E. coli* strain were captured using an Olympus 10× UPlanFL objective lens (0.30 NA; 824 µm × 824 µm field) or a 20× UCPlanFL objective lens (0.70 NA; 416 µm × 416 µm field) at 525 ± 45 nm (excitation) / 645 ± 75 nm (emission). The non‐fluorescent *E. coli* MG1655 and *S. marcescens* were imaged in bright‐field using an Olympus 40× LUCPlanFL objective lens (0.60 NA; 195 µm × 195 µm field). All images were focused on the glass substrate.

Time‐lapse videos were recorded every 30 min over 18 h. Each video contained 120 frames with 0.11s intervals. Videos were analyzed using Fiji‐Trackmate [15, 18, 46, 50]. Trajectories with < 10 µm displacements were removed. Only cells appearing in ≥30 consecutive frames were used for swimming‐speed calculations [18].

### 5.11 Statistical Analysis

For real‐time on chip experiments, at least two biological replicates were performed. For off‐chip experiments, at least three biological replicates were conducted. Unless noted, results were presented as mean ± standard deviation. Statistical analysis uses GraphPad Prism 8. Statistical significance was determined as *p* < 0.05.

## Author Contributions

**Jianan Yin**: investigation, writing – review and editing, methodology. **He Huang**: investigation, writing – review and editing. **Maggie Cusack**: investigation, writing – review and editing. **Andrew Glidle**: investigation, methodology, writing – review and editing. **Huabing Yin**: conceptualization, investigation, funding acquisition, writing – review and editing, methodology, validation, software, project administration, resources, supervision, data curation. **Xiaobo Li**: conceptualization, investigation, writing – original draft, methodology, validation, visualization, writing – review and editing, formal analysis, data curation. **Yanqing Song**: investigation, methodology, writing – review and editing. **William Sloan**: investigation, writing – review and editing.

## Conflicts of Interest

The authors declare no conflicts of interest.

## Supporting information




**Supporting File 1**: smll75309‐sup‐0001‐SuppMat.docx.

**Supporting File 2**: smll75309‐sup‐0002‐VideoS1.avi.

**Supporting File 3**: smll75309‐sup‐0003‐VideoS2.avi.

**Supporting File 4**: smll75309‐sup‐0004‐VideoS3.avi.

**Supporting File 5**: smll75309‐sup‐0005‐VideoS4.avi.

**Supporting File 6**: smll75309‐sup‐0006‐VideoS5.avi.

## Data Availability

All raw sequencing data for the original strain, mutant strains T‐10G‐1 and L‐20G‐1 have been deposited under the accession numbers SRX27262085, SRX27262086 and SRX27262087 respectively in the NCBI SRA database (https://www.ncbi.nlm.nih.gov/sra/PRJNA1207045).
