# Supplementary material for: Programmable 3D Microfluidics Reveals Motility‐Driven Antibiotic Resistance Evolution in Gradients
Source: Small. 2026 Aug 18;22(59):e75309. doi: 10.1002/smll.75309 (PMC13690616; doi:10.1002/smll.75309)
Supplement: Supplementary file 1 [file SMLL-22-e75309-s005.docx]

# Supplementary Information

**Programmable 3D Microfluidics Reveals Motility-Driven Antibiotic Resistance Evolution in Gradients**

Xiaobo Li ^a^, Jianan Yin ^b^, Yanqing Song ^a^, Andrew Glidle ^a^, William Sloan ^a^, He Huang ^b^, Maggie Cusack ^c^, Cindy J Smith ^a^, and Huabing Yin ^a^ *

^a^ James Watt School of Engineering, Advanced Research Centre (ARC), University of Glasgow, Chapel Lane, Glasgow G11 6EW, UK.

^b^ School of Synthetic Biology and Biomanufacturing, State Key Laboratory of Synthetic Biology, Tianjin Key Laboratory of Biological and Pharmaceutical Engineering, Tianjin University, Tianjin 300350, China

^c^ Munster Technological University, Rossa Avenue, Bishopstown, Cork, T12 P928,

Ireland

* Corresponding Author:

Huabing Yin; [huabing.yin@glasgow.ac.uk](mailto:huabing.yin@glasgow.ac.uk).

# 1. Experimental section

## 1.1. MIC Determination

A microdilution broth method was used to determine the minimum inhibitory concentrations (MICs) as recommended CLSI document M100 [1]. In brief, 100 μL of a two-fold serial dilution of antibiotic solutions was added to a 96-well plate containing 100 μL bacterial suspension (final bacterial number of ~ 5 × 10^5^ CFU/well), and then the plate was incubated for 20-24 h at 37°C in ambient air. The MIC was defined as the lowest concentration producing no visible growth. Cation-adjusted Mueller-Hinton broth (CAMHB) was used in all tests described in this section. Three technical replicates were performed for each condition.

## 1.2. Chip operation

After priming the chip, as illustrated in Figure S1b, the migration channel was first closed by injecting water at 10 μL/min into on-chip valve channel 1, thereby closing the lower layer migration channel. Antibiotic and bacterial solutions were then loaded into their respective reservoirs. Next, water was injected at 10 μL/min into on-chip valve channel 2 to close the inlet and outlet channels. This step is crucial to ensure that there is no flow within the system. The migration channel was then reopened by withdrawing water at 2 μL/min from on-chip valve channel 1. Bacterial migration was subsequently recorded within the observation window. At specific time points (e.g., 18 h), the migration channel was closed and the inlet and outlet channels were reopened. By sequentially introducing air bubbles followed by PBS solutions from the inlet channel, a high retrieval efficiency from both the antibiotic and bacterial reservoirs was achieved (Figure S9). This method was also sufficient for removing biofilms from the chip surface. The retrieved solutions were collected from the outlet channel into a 1.5 mL Eppendorf tube.

## 1.3. Gradient formation

A fluorescein solution (100 μM) was used to characterize the formation of concentration gradients in the migration channel. Due to its similar molecular weight to the antibiotics, fluorescein has a comparable diffusion coefficient. The operation was performed as described in Section 1.2, except that the antibiotic reservoir was replaced with fluorescein solution and the bacterial reservoir was filled with water. Once the migration channel was opened, time-lapse fluorescence imaging along the channel was recorded under a 4× objective lens with 470-495 nm excitation and 510-555 nm emission.

Antibiotics at different concentrations were loaded into the antibiotic reservoir. An antibiotic gradient was then formed along the migration channel and is referred to as an ×MIC antibiotic gradient in the manuscript. For example, a 10MIC GEN gradient indicates that 10MIC GEN was loaded into the antibiotic reservoir. Unless otherwise noted, the total exposure time was 18 h.

For time-course experiment (a), bacteria were exposed to the 10MIC gradient for 1, 5, 10, or 15 h, after which they were directly isolated from the chip. For time-course experiment (b), bacteria were exposed to the 10MIC gradient for 5, 10, or 15 h. The migration channel was then closed, thereby eliminating the antibiotic gradient. Cells that had migrated into the antibiotic reservoir were collected at 18 h.

To assess potential antibiotic adsorption in PDMS chips, 100MIC GEN, 100MIC CIP, or 100MIC AMP solutions were loaded into the chip system. After 18 h, the antibiotic solutions were collected and used to determine MIC values. Fresh stock solutions were used as controls. After 24 h of incubation, MIC values were measured, and bacterial growth rates were calculated using the following equation:

Growth rates (%) = (net OD_600nm_ with antibiotic / net OD_600nm_ without antibiotic) × 100%

## 1.4. Imaging of bacterial morphology under different antibiotics

The log-phase bacterial suspension was adjusted to OD_600nm_ = 0.2, then incubated in TB medium or treated with antibiotics at concentrations of 100xMIC GEN, 50xMIC AMP, or 10xMIC CIP at 37 °C for 2 hours. A 10 μL bacterial suspension was placed on a glass slide and covered with a coverslip. Fluorescence imaging of cells was performed using a 60× LUCPlanFL objective lens (0.75 NA) at 525 ± 45 nm (excitation) / 645 ± 75 (emission).

## 1.5. On-chip chemotaxis assays

The on-chip chemotaxis assays were performed as previously described [2]. In brief, bacteria were suspended in the chemotactic buffer (CB: 1× PBS, 0.1 mM EDTA, 10 µM L-methionine, and 10 mM DL-lactate. pH 7.0) at identical densities. After the migration channel was closed, the bacterial suspension was loaded into the reservoir, and the antibiotic solutions, chemotactic buffer (control), or 0.5 mM aspartic acid (chemoattractant) was loaded into the chemoattractant reservoir. Both the inlet and outlet channels were then closed to prevent convection. Time-lapse images were recorded every 30 minutes at the observation window after opening the migration channel.

## 1.6. Bacterial growth curves measurement

All growth curves were measured in TB unless comparing across TB, LB, and M9. A 100 μL bacterial suspension was added to each well of a 96-well plate, and incubated in a plate reader (Synergy HT, BioTek) at room temperature. OD_600nm_ was measured every 30 minutes for 22 hours. Three to five technical replicates were tested per condition.

## 1.7. Heteroresistance tests

The ancestral strain was grown in CAMHB medium to exponential phase and adjusted to OD _600nm_ = 0.2. Then, 100 μL of suspension was spread onto Mueller-Hinton agar (MHA) plates. A 10 μg GEN disk was placed at the centre of each plate and incubated at 37 °C for 24 hours. If no colonies appeared inside the inhibition zone, the strain was considered non-heteroresistant [3]. To test tolerance, 2 mg of glucose solution was added to the centre of the plate after removing the disk and incubated for an additional 24 hours. If no colonies emerged, the strain was considered non-tolerant to GEN [4].

## 1.8. Motility tests

Bacterial motility was assessed using imaging-based and soft agar assays:

1) Imaging-based motility assay.

Log phage bacteria (OD_600nm_ = 0.2) were diluted 50-fold in TB. A 100 μL bacterial suspension (with or without phenamil, 50 - 500 μM) was added into 96-well plates. Time-lapse fluorescence videos were taken using an Olympus 20 × UCPlanFL objective lens (0.70 NA) at 525 ± 45 nm (excitation) / 645 ± 75 (emission). Videos contained 60 frames with a 0.04 s interval. Trajectories with < 10 μm displacement were considered non-swimming cells or surface-attached. Swimming speeds of individual cells were calculated using Fiji-Trackmate. Twelve videos from three independent experiments were analysed per condition.

2) Soft-agar based swimming assay.

Soft agar (1% tryptone, 0.8% NaCl, 0.25% agar) plates were inoculated with motile strain (MG1655:: P_LacUV5_-mRFP) or non-motile strain (BL21:: P_EXP5_-RFP1) at equal density (OD_600nm_ = 1.0). Plates were incubated at 30 °C for 72 hours. Swimming diameters were measured every 24 hours. Six replicas were tested per strain.

## 1.9. Inhibition tests by phenamil

A 100 μL bacterial suspension was added into 96-well plates with or without phenamil. Plates were incubated at room temperature (about 22 °C) for 24 hours, followed by OD_600nm_ measurement. Net OD values were corrected using blank medium controls. Growth without phenamil was set to 100 %, and relative growth was calculated:

Relative growth (%) = (net OD_600nm_ with phenamil / net OD_600nm_ without phenamil) × 100%

## 1.10. Polymerase chain reaction (PCR) tests

Mutant strains (MIC ≥ 4 mg/L) isolated from chips were cultured to log phase. Cells were harvested and DNA was extracted using the rapid PBC method (Potassium hydroxide + boiling + centrifugation) [5]. PCR was used to amplify the gentamicin-resistant genes and *uspA*, using primers listed in Table S3. Reactions (50 μL) contained 2 μL DNA template, 1 μL forward primer (10 μM), 1 μL reverse primer (10 μM), 25 μL *Taq* 2× Master Mix (New England Biolabs, Catalog number: M0270), and 21 μL nuclease-free PCR-grade water. Cycling conditions: 95 °C for 5 min; 30 cycles of 95 °C for 30 s, 56.7-63.0 °C for 1 min, 68 °C for 1 min; and final extension at 68 °C for 5 min. PCR products were analysed on 2% agarose gel (electrophoresis) and imaged using a Syngene^TM^ system.

## 1.11 Intercellular ATP measurement

The gentamicin-resistant strains T-10G-14, T-10G-15, and L-20G-1, together with the ancestral strain, were cultured overnight in LB medium. Subsequently, 100 μL of each overnight culture was inoculated into 3 mL of fresh LB medium and incubated for another 4-6 h until the bacteria reached the exponential growth phase (OD_600nm_ = 0.3-0.6). All cultures were then adjusted to the same cell density (OD_600nm_ = 0.2) using fresh LB medium. A 100 μL aliquot of each bacterial suspension was mixed with 100 μL of BacTiter-Glo™ Reagent from the BacTiter-Glo™ Microbial Cell Viability Assay kit (Promega, G8230) and transferred to a white 96-well microplate. The plate was incubated on an orbital shaker for 5 min, after which luminescence was measured using a microplate reader (Synergy HT, BioTek). LB medium was used as the negative control. Three technical replicates were tested for each strain, and three independent biological replicates were performed.

## 1.12. Survival percentage assay

The survival percentage of bacteria in M9 salts medium supplemented with different carbon sources was determined following a previously reported method [6]. Briefly, the gentamicin-resistant strains T-10G-14, T-10G-15, and L-20G-1, together with the ancestral strain, were cultured in LB medium to the exponential growth phase. The bacterial cells were then washed three times with M9 salts medium (without any carbon source) and adjusted to an OD_600nm_ of 0.02 in 1 mL of M9 salts medium. Gentamicin was added at 0.5 × MIC (0.25 mg/L for the ancestral strain and 16 mg/L for the gentamicin-resistant strains). D-glucose (10 mM) or D-mannitol (10 mM) was added as the carbon source. Sodium acetate was added at 10 mM as a supplement and at 20 mM as a potentiating metabolite in the control experiments. The bacterial suspensions were incubated at 37 °C for 6 h. After incubation, the cultures were serially diluted and plated on LB agar plates to determine the number of colony-forming units (CFU/mL). Percentage survival was calculated as the ratio of bacterial density after 6 h of treatment to the initial density.

# 2. Figures

Figure S1. Microfluidic platform and operation. (a) Photographs of the mould (left) and a microfluidic device (right). Two on-chip valves control on/off of the migration and delivery channels. (b) The microfluidic chip consists of two separate reservoirs (~30 μL) for a bacterial suspension and an antibiotic solution, respectively. The reservoirs are linked by a migration channel (1500 μm length × 500 μm width × 100 μm maximum height) where an antibiotic gradient forms. The valve channels are separated from the lower migration channel by a thin 50 μm membrane. Time-lapse images were acquired from the observation window (indicated by the red dotted frame). Bacteria that migrated into the antibiotic reservoir were isolated at user-defined durations for off-chip characterisation. (c) A stable fluorescein gradient was established within approximately 50 min along the migration channel when the antibiotic reservoir was filled with fluorescein. Fluorescein was used as a proxy for antibiotic gradient formation along the migration channel. The spike in the curve is due to light scattering at the valve channel edge. BR: bacteria reservoir. AR: antibiotic reservoir. MC: migration channel.

Figure S2. Growth rates of bacteria under different antibiotics. Antibiotics collected from the microfluidic PDMS chip and the original stock solutions showed identical MIC values for all tested antibiotics (black arrows indicate the MIC) and similar growth rates across a range of antibiotic concentrations, indicating that antibiotic adsorption in the chip is minimal. CIP: ciprofloxacin; AMP: ampicillin; GEN: gentamicin.

Figure S3. Movement bias (β) of bacteria under different antibiotic gradients. Numbers before antibiotics indicate concentrations expressed as fold x MIC. TB: tryptone broth. CIP: ciprofloxacin. AMP: ampicillin. GEN: gentamycin.

| Cell ID | Swimming events |
| --- | --- |
| 1 | 0-10 min: stay in MC |
| 2 | 0min: stay in AR; 1min 1s: migrate into MC; 3min 15s: out of the observation window in MC |
| 3 | 1min: stay in AR; 1min 12s: migrate into MC; 4min 11s: migrate into AR; 5min 52s: out of observation window in AR |
| 4 | 2min: stay in MC; 4min 15s: migrate into AR; 5min 13s: attach the surface of AR d keep still until 10min. |
| 5 | 2min 5s: stay in MC; 3min 9s: migrate into AR; 4min 30s: migrate into MC; 5min 58s: migrate into AR; 6min 46s: migrate into MC; 9min 45s: migrate into AR;10min: stay in AR |

Figure S4. Bacterial movement events. Time-lapse images were recorded over 10 minutes after 8 h of migration. Each white dot represents a single cell. The channel edges were indicated by white dotted lines, while individual bacteria and their swimming trajectories were displayed in corresponding colours. Open dotted circles mark the starting points, and arrows indicate the endpoints of each trajectory. The table summarizes the swimming events of five individual cells. These bacteria displayed random movement between the migration channel (MC) and the antibiotic reservoir (AR), indicating that they were exposed to the antibiotic gradient for an extended period rather than transient interactions. Scale bar: 100 μm. MC: migration channel; AR: antibiotic reservoir.

Figure S5. Bacterial growth curve over time in TB at room temperature (~ 22 ℃). Cell numbers were counted using the dilution plating method on LB agar plates.

Figure S6. Number of cells in the migration channel within the observation window. After 10 h, cell numbers increased dramatically with time, making it difficult to identify individual cells. Therefore, swimming cells were tracked only within the first 10 h.

Figure S7. Fluorescence imaging of bacteria under a coverslip with different antibiotic treatments at 37 °C for 2 hours. Bacteria exhibited elongation when exposed to 10 MIC CIP or 50 MIC AMP. Scale bar: 20 μm. GEN: gentamycin. CIP: ciprofloxacin. AMP: ampicillin.

Figure S8. Number of cells and bacterial morphology within the migration channel at 18 h under TB, GEN, CIP, and AMP gradients.

Figure S9. Bacterial swimming trajectories in antibiotic reservoirs loaded with 100 MIC GEN, 10 MIC CIP, and 50 MIC AMP after 18 h of migration. Each single white dot represents a cell. Different colours indicated swimming trajectories of individual cells. For clarity, only a subset of trajectories is shown.

Figure S10. Retrieval efficiency**.** (a) The bacterial density after retrieval was comparable to the initial loading density. (b) A retrieval efficiency of more than 93% was achieved. Retrieval efficiency was defined as the ratio of the retrieved bacterial density to the initial loading density. (c) Following the sequential introduction of air bubbles and a PBS wash, dense biofilms were effectively removed and collected from both reservoirs. Only a small number of bacteria remained at the edges of the channel (white arrows).

Figure S11. Responses of bacteria in sub-MIC and antibiotic-free regions. (a) Representative images of the entire migration channel at 18 h. In the zoom-in images, bacterial filamentation was observed in the sub-MIC regions only under the 10MIC CIP and 10MIC AMP gradient conditions (yellow arrows indicated). (b) Schematic illustration of the isolation strategy used to collect bacteria from different regions of the microfluidic chip. (c) Number of colonies formed on LB agar and on LB agar containing the same antibiotic concentration as the reservoir from the retrieved cells. MC: migration channel. AR: antibiotic reservoir. BR: bacterial reservoir.

Figure S12. Characterisation of bacteria that migrated into the antibiotic reservoir (AR). (a) Representative images of the AR filled with 10 MIC GEN at different migration time points. Each white dot represents a cell. (b) Average number of bacteria in the AR per field (824 μm × 824 μm). Data were derived from at least 16 randomly selected images in the AR across two biological replicates. The red arrows indicate the time point at which successful migration occurred (i.e., > 1000 swimming cells were tracked in the observation window). AR: antibiotic reservoir.

Figure S13. (a) Migration curve (i.e. number of cells per field versus time) when both reservoirs were loaded with 10 MIC GEN. Grey dotted lines indicate the threshold of 1000 swimming cells per field of view. (b) No colonies formed from cells collected after 18 h of migration. The dotted grey lines indicate the detection limit. (c) Assessments of resistance formation in the absence of a gradient. The ancestral strain was inoculated into TB, either alone or supplemented with 10 MIC GEN (5 mg/L) with a final cell density of approximately 10⁷ cells/mL and incubated at room temperature for 18 h. After incubation, 100 μL of the suspension was plated onto LB agar to assess viability. Scale bar: 10 mm. LBA: LB agar. GEN: gentamicin. ND: not detected.

Figure S14. Growth curves of the ancestral strain under various loading densities. Dotted lines indicate the time points at which successful migration occurred in Figure 5a, namely 9 h, 16 h and 25 h for 10^7^ cells/mL, 10^6^ cells/mL, 10^5^ cells/mL, respectively. A similar OD_600_ value (approximately 0.11) was reached at these time points across all three loading densities.

Figure S15. Growth curves under different media. M9: minimal 9 medium. TB: tryptone broth. LB: lysogeny broth. Bacteria did not exhibit any measurable growth when cultured in M9 medium for 22 hours.

Figure S16. Growth curves of the ancestral strain in TB with or without 4MIC GEN.

Figure S17. Inhibition of bacterial motility by phenamil. (a) Growth rates of bacteria under phenamil treatment. (b) Percentage of swimming bacteria in the total population after treatment with different concentrations of phenamil. (c) Bacterial swimming speeds under phenamil treatments in well-plates. In total, 200 cells were analyzed per condition, except for the 500 μM phenamil group, where 110 cells were analysed. All speeds were normalized to the corresponding control groups. Data in (b) and (c) were from swimming assays performed in well plates. (d) Swimming motility on soft agar for a motile strain and a non-motile strain after 48 h of incubation at 30 ℃. Scale bar: 10 mm. (e) Swimming diameters of the motile and non-motile strains at different incubation times. Unlike the motile strain, the non-motile strain exhibited no swimming ability. Statistics: Kruskal Wallis with Dunn’s post-test. ns: not significant; *p< 0.05, **p< 0.01, ***p< 0.001, ****p< 0.0001.

Figure S18. Heteroresistance test. The white dotted circles indicate inhibition zones. No colonies were detected in the inhibition zone before removal of the GEN disk or after glucose addition.

Figure S19. MICs of viable bacterial strains randomly selected from LB agar containing 5MIC GEN. These cells were collected after 18 h of migration under 5MIC GEN gradients in TB medium. Grey dotted lines indicate the original MIC and susceptibility breakpoints. MIC: minimum inhibitory concentration. S: susceptible. I: intermediate. R: resistant.

Figure S20. Agarose gel images for gentamicin-resistant genes for (a) *aad B* (700 bp), (b) *aac(6′)-Ie-aph(2′)-Ia* (369 bp), (c) *aac(3)-VI* (319 bp), (d) *aacC2* (740 bp), and (e) *aac(3)-IV* (653 bp). (f) The gene *uspA* (884 bp) was used as a target for the detection and verification of *E. coli*. M: DNA marker, specific base pairs (bp) were marked. Lane 1-8 for strains T-10G-2, T-10G-3, T-10G-19, T-10G-20, T-10G-9, T-10G-14, T-10G-15, and original strain, respectively.

Figure S21. MICs of gentamicin-resistant strains compared with the original strain. MICs for GEN increased only in these mutant strains T-10G-14, T-10G-15 and L-20G-1, while MICs for CIP and AMP remained unchanged compared to the original strain, indicating that the resistance mechanism is specific to GEN. GEN: gentamicin. CIP: ciprofloxacin. AMP: ampicillin.

# 3. Tables

Table S1. Components of the three media.

| **M9 minimal medium (M9)** | | **Tryptone broth (TB)** | | **Lysogeny broth (LB)** | |
| --- | --- | --- | --- | --- | --- |
| **Ingredients** | **g/L** | **Ingredients** | **g/L** | **Ingredients** | **g/L** |
| Na_2_HPO_4_ | 6 | Tryptone | 10.0 | Peptone | 10.0 |
| KH_2_PO_4_ | 3.0 | NaCl | 8.0 | NaCl | 5.0 |
| NaCl | 0.5 |  |  | Yeast Extract | 5.0 |
| NH_4_Cl_2_ | 1 |  |  |  |  |
| Glucose | 4 |  |  |  |  |
| MgSO_4_ | 0.12 |  |  |  |  |
| CaCl_2_ | 0.011 |  |  |  |  |

Table S2. Total variants detected in gentamicin-resistant strains.

| **Strains** | **Position** | **Gene** | **SNP/Indel** | **Reference** | **Alteration** |
| --- | --- | --- | --- | --- | --- |
| T14 | 1209618 | *stfE* | Indel | A | AGGTTTTCGAGAGC |
|  | 1209622 | *stfE* | Indel | T | TTGCACAGTGCCATCCGATTTGATATCGCCAAACGGATTCTTGCGGC |
|  | 1978502 | *-* | Indel | G | GTATAAGTCATACTTTTGTTTTGGGTGTATTTCAATCTGTTAAAAAGTTTTTCGCTACGCTAGCAA |
|  | 3214775 | *rpoD* | SNP | G | A |
|  | 3917527 | *atpG* | Indel | TC | T |
|  | 3982841 | *-* | Indel | T | TCCTAGGTTGTGTCTCAAAATCTCTGATGTTACATTGCACAAGATAAAAATATATCATCATGAACAATAAAACTGTCTGCTTACATAAACAGTAATACAAGGGGTGTTATGAGCCATATTCAGCGTGAAAC |
| L21 | 1209618 | *stfE* | Indel | A | AGGTTTTCGAGAGC |
|  | 1209622 | *stfE* | Indel | T | TTGCACAGTGCCATCCGATTTGATATCGCCAAACGGATTCTTGCGGC |
|  | 366379 | *-* | Indel | A | ACG |
|  | 366381 | *-* | Indel | C | CCTCCTGTGTGAAATTGTTATCCGCTTTAATTAAAGGCATCAAATAAAACGAAAGGCTCAGTCGAAAGA |
|  | 3916337 | *atpD* | Indel | G | GGTCCAGCTGACGGCTGGTGGA |
|  | 3917537 | *atpG* | Indel | ACGGG | A |
|  | 3920789 | *atpF* | SNP | C | A |

Indel: insertion and deletion. SNP: Single-nucleotide polymorphism.

Table S3. Primers used in this study.

| **Target gene(s)** | **PCR primer sequence (5'–3')** | **Length (bp)** | **T_m_ (℃)** | **References** |
| --- | --- | --- | --- | --- |
| *aadB*  *(*or *ant(2”)-Ia)* | F: TCCAGAACCTTGACCGAAC  R: GCAAGACCTCAACCTTTTCC | 700 | 57.3 | [7] |
| *aac(6**′)-Ie-aph(2′)-Ia* | F: CAGGAATTTATCGAAAATGGTAGAAAAG  R: CACAATCGACTAAAGAGTACCAATC | 369 | 63.0 | [8] |
| *aac(3)-VI* | F: TATTCTCGCCTTCGTCTCGT  R: GCCGATCGACAGGATTTTT | 319 | 57.3 | [9] |
| *aacC2*  *(*or *aac(3)-IIa)* | F: CGGAAGGCAATAACGGAG  R: TCGAACAGGTAGCACTGAG | 740 | 56.7 | [7] |
| *aac(3)-IV* | F: TGCTGGTCCACAGCTCCTTC  R: CGGATGCAGGAAGATCAA | 653 | 61.4 | [9] |
| *uspA* | F: CCGATACGCTGCCAATCAGT  R: ACGCAGACCGTAAGGGCCAGAT | 884 | 59.0 | [10] |

F: forward primer, R: reverse primer.

# 4. Videos

Video 1. Motile *E. coli* exhibiting random migration in the 10MIC GEN gradient.

Video 2. *E. coli* bacteria maintaining active motility in the 100MIC GEN reservoir.

Video 3. *E. coli* bacteria displaying low motility in the 100MIC CIP reservoir.

Video 4. *E. coli* bacteria displaying low motility in the 100MIC AMP reservoir.

Video 5. *S. marcescens* bacteria exhibiting random migration in the 10MIC GEN gradient and achieved successful migration at around 9h.

# References

1.	CLSI. Performance standards for antimicrobial susceptibility testing. 33th ed. 2023

2.	Li X, Song Y, Glidle A *et al.* A simple three-dimensional microfluidic platform for studying chemotaxis and cell sorting. *Lab Chip*. 2025 <https://doi.org/10.1039/d4lc00892h>

3.	El-Halfawy OM, Valvano MA. Antimicrobial heteroresistance: An emerging field in need of clarity. *Clin. Microbiol. Rev*. 2015;**28**:191-207 <https://doi.org/10.1128/cmr.00058-14>

4.	Li X, Song Y, Chen X *et al.* Single-cell microfluidics enabled dynamic evaluation of drug combinations on antibiotic resistance bacteria. *Talanta*. 2023 <https://doi.org/10.1016/j.talanta.2023.124814>

5.	Liu Y, Chen J, Cheng Y *et al.* A simple and rapid technique of template preparation for pcr. *Front. Microbiol*. 2022;**13** <https://doi.org/10.3389/fmicb.2022.1024827>

6.	Allison KR, Brynildsen MP, Collins JJ. Metabolite-enabled eradication of bacterial persisters by aminoglycosides. *Nature*. 2011;**473**:216-20 <https://doi.org/10.1038/nature10069>

7.	Soleimani N, Aganj M, Ali L *et al.* Frequency distribution of genes encoding aminoglycoside modifying enzymes in uropathogenic e. Coli isolated from iranian hospital. *BMC Res. Notes*. 2014;**7**:842 <https://doi.org/10.1186/1756-0500-7-842>

8.	Vakulenko SB, Donabedian SM, Voskresenskiy AM *et al.* Multiplex pcr for detection of aminoglycoside resistance genes in enterococci. *Antimicrob. Agents Chemother*. 2003;**47**:1423-26 <https://doi.org/10.1128/aac.47.4.1423-1426.2003>

9.	Chalmers G, Cormier AC, Nadeau M *et al.* Determinants of virulence and of resistance to ceftiofur, gentamicin, and spectinomycin in clinical escherichia coli from broiler chickens in quebec, canada. *Vet Microbiol*. 2017;**203**:149-57 <https://doi.org/10.1016/j.vetmic.2017.02.005>

10.	Chen J, Griffiths MW. PCR differentiation of escherichia coli from other gram-negative bacteria using primers derived from the nucleotide sequences flanking the gene encoding the universal stress protein. *Lett. Appl. Microbiol*. 1998;**27**:369-71 <https://doi.org/10.1046/j.1472-765x.1998.00445.x>
